# Uncoupling TGFβ1 signalling from collagen protein synthesis in Dupuytren's disease

**DOI:** 10.1002/path.70020

**Published:** 2026-01-26

**Authors:** Gabriella Cooper, Jade A Gumbs, Sahem Alkharabsheh, Katie J Lee, Alan Carter, Hannah Coleman, Niamh S O'Heneghan‐Yates, Rama Ijaz, Emma Beamish, Lisa A Menezes, Triantafillos Liloglou, Peter D Clegg, Elizabeth G. Canty‐Laird

**Affiliations:** ^1^ Department of Musculoskeletal and Ageing Science, Institute of Life Course and Medical Sciences University of Liverpool Liverpool UK; ^2^ The Medical Research Council Versus Arthritis Centre for Integrated Research into Musculoskeletal Ageing (CIMA) Liverpool UK; ^3^ Faculty of Allied Medical Sciences, Department of Medical Laboratory Science Mutah University Mu'tah Jordan; ^4^ Edge Hill University Ormskirk UK

**Keywords:** collagen, fibrosis, TGF‐beta, Dupuytren's, metabolic labelling, proteomics

## Abstract

Dupuytren's disease is a fibroproliferative disorder of the palmer fascia (PF) characterised by flexion contractures in the hand. Dupuytren's disease can be treated surgically, but disease recurrence rates are high, potentially due to continual production of matrisomal proteins. Here, metabolic labelling and proteomics identified differences in the new synthesis and composition of matrisomal proteins between Dupuytren's tissue and normal PF. Dupuytren's tissue actively synthesised type I collagen, fibronectin (FN1), matrix metalloproteinases‐2 and ‐3 (MMP2, MMP3) and tissue inhibitor of metalloproteinases 2 (TIMP2). Both tissues actively synthesised insulin‐like growth factor binding protein 7 (IGFBP7). Label‐free analysis implicated the transforming growth factor‐β (TGFβ) pathway in the matrisomal profile of Dupuytren's tissue. The effect of TGFβ isoforms on *COL1* mRNA expression was first tested in cultured young and aged equine tenocytes. *COL1A1* mRNA responded to treatment with all TGFβ isoforms and was more highly expressed in cells from aged samples. In aged human cells, *COL1A1* and *COL1A2* mRNA was higher in cells derived from Dupuytren's tissue than normal PF and in response to TGFβ1, but no changes in *COL1A1* or *COL1A2* CpG methylation were detected. TGFβ1 treatment only resulted in increased type I collagen protein accumulation in the media of Dupuytren's nodule cells. In three‐dimensional cultures, *COL1A1* mRNA was lower in normal PF than in Dupuytren's cells, but TGFβ1 treatment only increased type I collagen accumulation in the media of normal PF cultures, and TGFβ1 inhibition did not alter new collagen protein synthesis. TGFβ1 inhibition in Dupuytren's tissue explants did not alter the proportion of homotrimeric type I collagen, nor was this changed in skin or tendon of the tight‐skin (TSK) mouse, a naturally occurring model of indirect TGFβ1 activation. Therefore, the role of TGFβ in Dupuytren's disease may be predominantly related to myofibroblast phenoconversion and contractility rather than directly altering collagen protein synthesis. © 2026 The Author(s). *The Journal of Pathology* published by John Wiley & Sons Ltd on behalf of The Pathological Society of Great Britain and Ireland.

## Introduction

Dupuytren's disease is a progressive fibroproliferative condition of the palmar fascia (PF) (aponeurosis) of the hand that can result in debilitating digital contractures [1, 2]. Following the formation of highly cellular ‘nodules’, collagenous cords align along the length of the fascia form and contract due to myofibroblast activity [3]. Progression is staged from 0 to IV depending on the degree of digit contracture [4]. Fibrosis is defined by the excessive accumulation of extracellular matrix (ECM) components leading to the formation of permanent fibrotic scar [5]. Dupuytren's disease pathogenesis involves genetic, cellular, and immunological factors [6]. Nodules are thought to represent the ‘active’ disease state [7] and are associated with high localised myofibroblast cell populations [3]. Myofibroblasts produce ECM secreting components, such as collagen and FN1 [8]. Myofibroblast maturation, marked by α‐smooth muscle actin expression, leads to a highly contractile cell phenotype that facilitates ECM tensioning via focal adhesions, resulting in digit contracture [9, 10].

Dupuytren's disease has a world‐wide prevalence of 8% and is associated with advancing age, male sex, family history, diabetes mellitus, heavy alcohol consumption, smoking, and a history of manual work [11, 12]. Although Dupuytren's disease has no cure, available treatments include surgery (primarily limited fasciectomy), collagenase or steroid injections, radiotherapy, or physiotherapy, depending on the disease stage [13]. Surgery and collagenase injections are associated with complications, and recurrence occurs in up to 50% of patients [14, 15, 16]. Understanding the ECM composition and key regulatory factors in Dupuytren's tissue that may contribute to disease recurrence could help identify targets for anti‐fibrotic treatments that offer more prolonged and effective disease management.

The ECM of fibrotic Dupuytren's tissue comprises abundant type I and type III collagens, including type I collagen homotrimer, FN1, proteoglycans, and periostin [9, 17, 18]. An altered matrix remodelling profile is suggested by altered mRNA expression of matrix metalloproteinases, a disintegrin and metalloproteinase domain with thrombospondin motif proteins, and tissue inhibitors of metalloproteinases in Dupuytren's cells or tissue [19, 20]. *MMP14* variants have also been genetically linked to Dupuytren's disease [21], and TIMP‐1 and ‐2, as well as MMP‐1, have been identified by immunohistochemistry in Dupuytren's tissue [22]. This suggests that Dupuytren's disease patients may have an altered matrisomal profile, promoting disease reoccurrence.

Proteomic profiling of Dupuytren's tissue has identified alterations in cytoskeletal proteins, as well as proteins involved in extracellular and intracellular signalling, oxidative stress, and cellular metabolism, along with activation of the Akt signalling pathway [23]. More recently, a tissue decellularisation approach in combination with proteomics identified extracellular fibrillar collagens, small leucine‐rich repeat proteoglycans, elastic fibre components, periostin, and laminins as more abundant in Dupuytren's tissue compared to normal PF [24]. ECM derived from Dupuytren's tissue promotes macrophage‐to‐myofibroblast transition, fibroblast migration, and the production of IL‐6, IL‐10, and TNF. Cytokines previously implicated in Dupuytren's disease include TNFα, TGFβ, IL‐6, IL‐10, IL‐1β, bFGF, and VEGF [25, 26, 27, 28].

TGFβ induces signalling through small mothers against decapentaplegic (SMAD)‐dependent (canonical) and SMAD‐independent (non‐canonical) pathways and can induce a wide range of physiological and pathological cellular effects [29]. TGFβ can drive fibrosis via fibroblast‐to‐myofibroblast conversion and overproduction of collagenous ECM [30]. Myofibroblast contractile force can further activate latent TGFβ1, inducing a positive feedback loop [31]. TGFβ present within Dupuytren's nodules and surrounding tissue may reflect interactions between TGFβ and fibroblasts, leading to further fibroblast activation and disease progression [26, 27]. While TGFβ leads to increased collagen synthesis [32, 33, 34, 35, 36], its direct effects on collagen synthesis may be confounded with global alterations in the fibroblast phenotype.

Here, we hypothesised that Dupuytren's disease would exhibit an altered ECM synthesis profile that contributes to disease recurrence. We utilised metabolic labelling in combination with proteomics and label‐free analysis to detect newly synthesised and accumulated matrisomal ECM proteins in Dupuytren's tissue. After identifying TGFβ1 as a predicted regulator of the matrisomal profile, the effect of modulating TGFβ on type I collagen mRNA expression and protein synthesis in two‐dimensional (2D) culture, three‐dimensional (3D) tissue‐like constructs, and tissue was determined.

## Materials and methods

### Ethical approval and patient consent

Human Dupuytren's and normal PF samples were obtained under UK National Health Service (NHS) Health Research Authority (HRA) Research Ethics Committee (REC) approval (REC13/NW/0352 [approved 22 May 2013, transfer to REC14/NW/0162 approved 11 July 2018] and REC 14/NW/0162 [approved 14 April 2014, ended 31 December 2023]), being gifted with full informed written patient consent from patients undergoing surgery at the Warrington and Halton Hospitals NHS Foundation Trust (now Warrington and Halton Teaching Hospitals NHS Foundation Trust) for Dupuytren's contracture or carpal tunnel syndrome respectively. The University of Liverpool was the named study sponsor, and the study was carried out in accordance with the Declaration of Helsinki (https://www.wma.net/policies-post/wma-declaration-of-helsinki/, date last accessed 4 December2025).

### Metabolic labelling with ^13^C_6_ ‐L‐lysine and mass spectrometry

Dupuytren's (*n* = 4) and normal PF (*n* = 5) (Table 1) tissue dissections were performed aseptically in DMEM (Thermo Fisher Scientific, Waltham, MA, USA) containing penicillin/streptomycin (1% v/v) (Thermo Fisher Scientific). Dupuytren's samples were separated into cord and nodule after fatty tissue removal and cut into pieces with a wet weight of ~25–50 mg or volume of ~25–50 μl. Normal PF samples were cut into two pieces of a similar size. One or two tissue pieces were equilibrated in 1 ml complete unlabelled DMEM for stable isotope labelling in cell culture (SILAC) medium (Sigma‐Aldrich, St. Louis, MO, USA) for 30 min at 37 °C, 5% CO_2_, and then in 1 ml complete [^13^C_**6**_]L‐lysine (Thermo Fisher Scientific) labelled SILAC medium [37] for 18–24 h. Explants were weighed, snap frozen, and stored at −80 °C, and medium was stored in aliquots at −20 °C. Details of sample preparation for mass spectrometry are described in the Supplementary materials and methods. Liquid chromatography tandem mass spectrometry (LC–MS/MS) was performed as previously described [37], with loading volumes determined from ranging runs. Details of the proteomic data analysis is provided in the Supplementary materials and methods.

**Table 1:** Human surgical samples used for proteomics, cell culture, and treatments.

Sample ID	Age (years)	Gender	Tissue type	Experiment	Data shown in figures
160,914‐51‐F	51	F	Normal PF	Proteomics	1, 2, S2, S3, S4, S5, S6, S7
161,005‐89‐F	89	F	Normal PF	Proteomics	1, 2, S2, S3, S4, S5, S6, S7
161,024‐48‐M	48	M	Normal PF	Proteomics	1, 2, S2, S3, S4, S5, S6, S7
161,024‐58‐M	58	M	Normal PF	Proteomics	1, 2, S2, S3, S4, S5, S6, S7
170,717‐68‐M	68	M	Normal PF	Proteomics	1, 2, S2, S3, S4, S5, S6, S7
160,912‐49‐M	49	M	Dupuytren's cord and nodule	Proteomics	1, 2, S2, S3, S4, S5, S6, S7
160,912‐75‐M	75	M	Dupuytren's cord and nodule	Proteomics	1, 2, S2, S3, S4, S5, S6, S7
160,928‐76‐M	76	M	Dupuytren's cord and nodule	Proteomics	1, 2, S2, S3, S4, S5, S6, S7
161,012‐62‐F	62	F	Dupuytren's cord and nodule	Proteomics	1, 2, S2, S3, S4, S5, S6, S7
161,012‐62‐F	62	F	Dupuytren's cord and nodule	Cell treatments	4, 5, S10, S11, S12
161,109‐70‐M	70	M	Dupuytren's cord and nodule	Cell treatments	4, 5, S10, S11, S12
161,130‐65‐M	65	M	Dupuytren's cord and nodule	Cell treatments	4, 5, S10, S11, S12
161,130‐76‐M	76	M	Dupuytren's cord and nodule	Cell treatments	4, 5, S10, S11, S12
170,201‐67‐M	67	M	Normal PF	Cell treatments	4, 5, S10, S11, S12
170,206‐69‐M	69	M	Normal PF	Cell treatments	4, 5, S10, S11, S12
170,301‐61‐F	61	F	Normal PF	Cell treatments	4, 5, S10, S11, S12
170,313‐57‐F	57	F	Normal PF	Cell treatments	4, 5, S10, S11, S12
170,403‐68‐M	68	M	Dupuytren's cord and nodule	Tissue treatments	6A
170,403‐86‐M	86	M	Dupuytren's cord and nodule	Tissue treatments	6A
170,412‐51‐M	51	M	Dupuytren's nodule	Tissue treatments	6A
170,412‐68‐F	68	F	Dupuytren's nodule	Tissue treatments	6A
170,508‐76‐M	76	M	Dupuytren's cord and nodule	Tissue treatments	6A
170,515‐65‐M	65	M	Dupuytren's cord and nodule	Tissue treatments	6A
170,802‐80‐M	80	M	Dupuytren's nodule	Tissue treatments	6A
170,816‐73‐F	73	F	Dupuytren's cord and nodule	Tissue treatments	6A
170,628‐55F	55	F	Normal PF	Western blotting	S8
170,809‐55 M	55	M	Normal PF	Western blotting	S8
170,814‐70 M	70	M	Normal PF	Western blotting	S8
180,514‐68F	68	F	Normal PF	Western blotting	S8
170,403–68 M	68	M	Dupuytren's cord and nodule	Western blotting	S8
170,403–86 M	86	M	Dupuytren's cord and nodule	Western blotting	S8
170,412–68F	68	F	Dupuytren's cord and nodule	Western blotting	S8
180,514‐60F	60	F	Dupuytren's	Western blotting	S8

Figures S2–S12 are provided in the supplementary material.

### Cell and tissue treatments

The experimental design is shown in the supplementary material, Figure S1, and human sample information in Table 1. Isolation and 2D and 3D culture of normal PF (*n* = 4) and Dupuytren's cells (*n* = 4) were carried out as previously described [18]. Equine tendon was obtained from an abattoir and tenocytes isolated from the mid‐metacarpal region of the superficial digital flexor tendon (SDFT) from young (*n* = 5, 4–7 years) and aged (*n* = 5, 15–28 years) cadavers as previously described [38], with the exception that 275 U/ml of collagenase (Worthington Biochemical Corporation, Lakewood, NJ, USA) was used for tissue digestion, and cells were split 1:2 with the same medium as for human cells. Equine tenocytes were treated at passage 1.

TGFβ stimulation or inhibition experiments were carried out as previously described for TNFα [18], except that treatments were carried out without serum. Equine tenocytes were treated with TGFβ1, TGFβ2, or TGFβ3 (Peprotech, Cranbury, NJ, USA) (1 or 10 ng/ml) or with a serum‐free control containing an equal volume of vehicle (10 mm citric acid pH 3.0) (Sigma‐Aldrich) for 24 h (*n* = 5 each for young and aged), or with TGFβ1 (1 or 10 ng/ml) for up to 48 h (0, 0.5, 1, 4, 24, and 48 h) including matching serum‐free controls (*n* = 4 each for young and aged). Human cells in 2D culture were treated with TGFβ1 (1 or 10 ng/ml), a matching serum‐free control, or with medium containing 10% foetal bovine serum (FBS) (Sigma‐Aldrich, F7524, Batch 024 M3398) for 16–18 h. Three‐dimensional cultures were treated with TGFβ1 (10 ng/ml) or a matching serum‐free control. Three‐dimensional cultures and tissue explants were treated with SD208 (Tocris, Bristol, UK) (1 μm from a 1 mm stock) with DMSO vehicle control. Radiolabelling with [^14^C]proline was carried out as previously described [18], with separate nodule (*n* = 8) and cord (*n* = 5) for Dupuytren's tissue explants. Western blotting of Dupuytren's (*n* = 4) and normal PF tissue extracts (*n* = 4) is described in the Supplementary materials and methods.

### Reverse‐transcription quantitative polymerase chain reaction (RT‐qPCR)

RNA extraction and RT‐qPCR were carried out for 3D constructs as previously described [18]. For 2D cultured cells, RNA extraction, purification, and cDNA synthesis were performed as previously described [39]. RT‐qPCR was then performed as reported [40], with 4 ng/μl cDNA template per reaction. Primer sequences are listed in the supplementary material, Table S1, and detailed methods are provided in the Supplementary materials and methods. Pyrosequencing is also described in the Supplementary materials and methods.

### Extraction and SDS PAGE analysis of salt‐soluble collagen

Surplus WT (*n* = 4) and heterozygous TSK mouse (*n* = 4) tail tendon and shaved dorsal skin were obtained from fresh 26‐week‐aged cadavers of a colony maintained in compliance with the Animals (Scientific Procedures) Act 1986 (project licence P267B91C3) and UK Home Office guidelines [41] and snap‐frozen. Acid‐soluble collagen was extracted from tissue samples with at least 10 volumes of 0.5 m acetic acid (Sigma‐Aldrich) containing protease inhibitors (complete protease inhibitor cocktail, Roche, Basel, Switzerland) for 18–24 h at 4 °C. Supernatants were precipitated with 0.8 m NaCl (Sigma‐Aldrich) in 0.5 m acetic acid for at least 18 h at 4 °C, to separate collagen types I–III [42] and pellets resuspended in 100 mm Tris–HCl (Sigma‐Aldrich). Extracts were analysed by electrophoresis on 6% Tris‐glycine gels (Thermo Fisher Scientific) with delayed reduction [43]. Gels were fixed with 10% methanol (Sigma‐Aldrich), 7% acetic acid overnight, stained with Bio‐safe Coomassie (Bio‐Rad, Hercules, CA, USA), destained with water and imaged at 700 and 800 nm using an Odyssey CLx Imager (LI‐COR, Lincoln, NB, USA) [44]. This method was chosen for its high linear dynamic range [45]. Relative quantification of the α1(I) and α2(I) chains was performed using Empiria software (LI‐COR).

### Statistical analyses

Data analysis was performed using SigmaPlot version 14.0 (Systat, Santa Clara, CA, USA) and graphing with GraphPad Prism 8 or 9 for Windows (GraphPad Software, La Jolla, CA, USA, www.graphpad.com), unless otherwise stated. Plots show individual data points with mean and ±1 SD unless otherwise indicated. Data were analysed by two‐way ANOVA and a Holm–Sidak *post hoc* test for two factors, using one‐way ANOVA for one factor, with Student's or Welch's *t*‐test, or with a one‐sample *t*‐test for comparison to a single value. A prior log transformation was applied to human RT‐qPCR data, or a suitable Box‐Cox transformation was used if assumptions of normality and equal variance (assessed with the Shapiro–Wilk and Brown–Forsythe tests respectively) were not met; otherwise, one‐way ANOVA on ranks with a Dunn's *post hoc* test was used. A *p* < 0.05 was considered statistically significant. Principal component analysis (PCA) and graphing were carried out using Minitab version 18 (Minitab LLC, State College, PA, USA) as were Box‐Cox transformations. Time course data were analysed with a two‐way repeated measures ANOVA in GraphPad Prism. For *p* values see the supplementary material, Table S4. Baye's factors were generated using an online web Bayes factor calculator (https://users.sussex.ac.uk/~dienes/inference/Bayes.htm, date last accessed 4 December 2025) with a uniform age prior and bounds of 75 (upper) and −75 (lower) [46].

## Results

### Active synthesis of matrisomal proteins by Dupuytren's tissue

**Figure 1:**
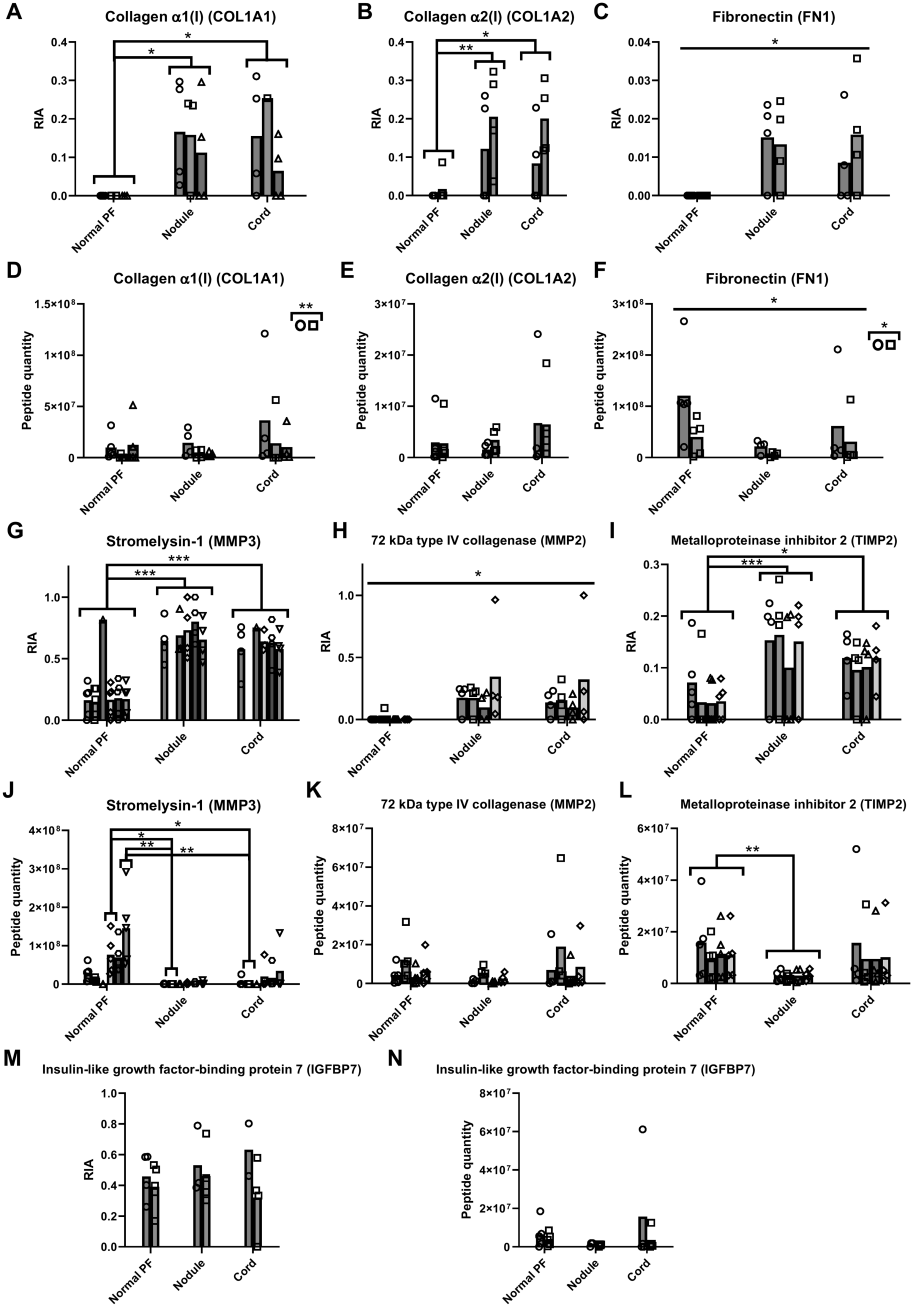
Labelled matrisomal proteins in medium of normal PF and Dupuytren's tissue explants. ECM proteins labelled with ^13^C lysine were identified using MASCOT and extracted ion chromatograms were analysed using Xcalibur. Heavy (H) and light (L) peaks were identified for each labelled peptide and the area under the peak recorded. (A–C,G–I,M) Relative isotope abundance (RIA) (H/(L + H)) and (D–F,J–L,N) total peptide quantity (H + L) normalised to equivalent tissue weight analysed are shown. (A,D) Collagen α1(I) (COL1A1), (B,E) collagen α2(I) (COL1A2), (C,F) fibronectin (FN1), (G,J) matrix metalloproteinase 3 (MMP3), (H,K) matrix metalloproteinase 2 (MMP2), (I,L) tissue inhibitor of metalloproteinase 2 (TIMP2), (M,N) insulin‐like growth factor‐binding protein‐7 (IGFBP7). Different symbols represent different peptides, and the bar represents the mean. *p < 0.05, **p < 0.01, and ***p < 0.001. *P* values for all significant comparisons are shown in the supplementary material, Table S4. Sample details are given in Table 1.

There were no significant differences in the age of control and Dupuytren's samples used for proteomics analysis (*p* = 0.796) (Table 2). Bayesian analysis indicated substantial evidence of no difference in age between the groups (*B* = 0.17). Tissue explants were labelled with heavy lysine, and both tissue extracts and media were analysed for new protein synthesis (heavy peptide content) and turnover (total peptide content) as previously described [37]. No labelling was consistently detected above background in tissue extracts. In media, labelled matrisomal proteins type I collagen, FN1, MMP3, MMP2, TIMP2, and IGFBP7 were detected (Figure 1), with labelled type I collagen, MMP3, and TIMP2 being significantly higher in media from Dupuytren's nodule (COL1A1; *p* = 0.022, COL1A2; *p* = 0.008, MMP3; *p* ≤ 0.001, TIMP2; *p* = 0.001) or cord (*p* = 0.023, *p* = 0.015, *p* ≤ 0.001, *p* = 0.031 respectively). Peptide quantity was not lower in normal PF medium, indicating that results were not skewed by explant size. The relative isotope abundance for FN1 was low (<5%), and IGFBP7 was actively synthesised by both control and Dupuytren's samples.

**Table 2:** Age and gender distribution of samples analysed by proteomics, with disease stage information for Dupuytren's samples (Tubiana classification [4]).

	Normal PF		Dupuytren's		
	Age (years)	Gender	Age (years)	Gender	Disease stage
	48	M	49	M	4
	51	F	62	F	2
	58	M	75	M	4
	68	M	76	M	2
	89	F			
Mean	62.8		65.5		
SD	16.5		12.7		

### TGFβ1 is implicated in the matrisomal profile of Dupuytren's tissue

**Figure 2:**
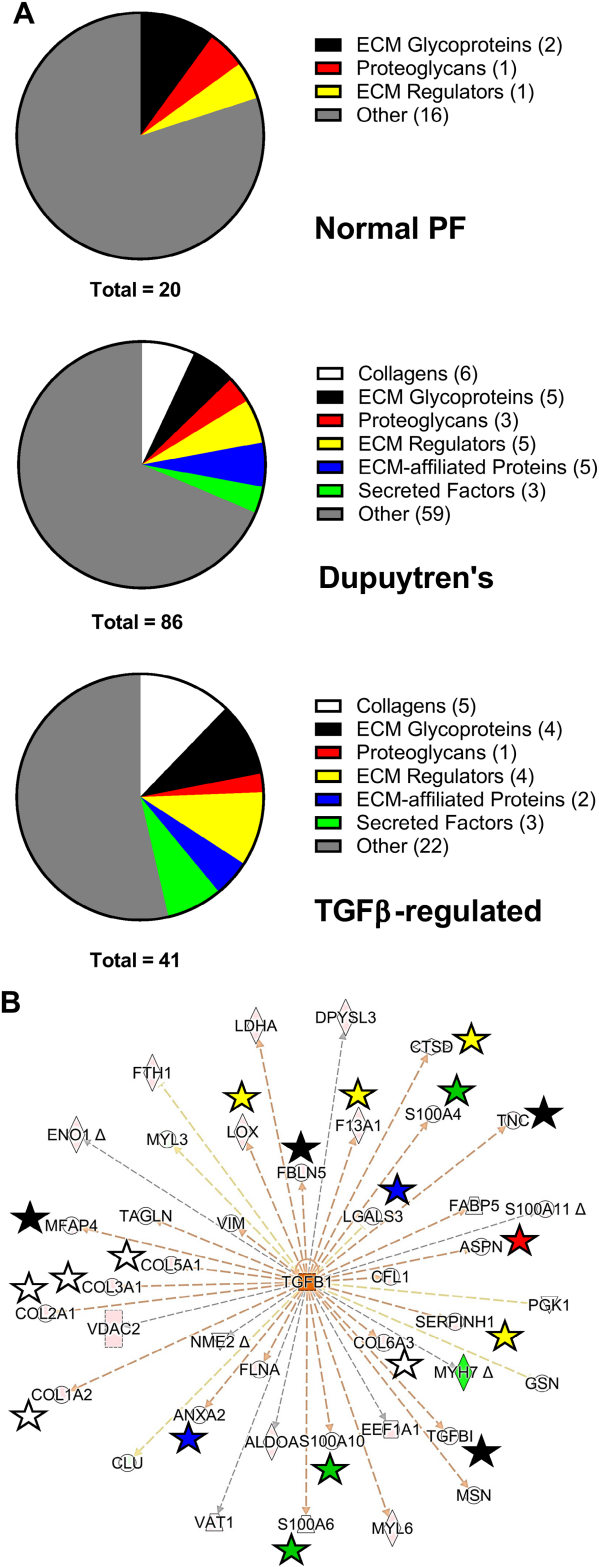
Matrisomal enrichment in the normal PF and Dupuytren's explant tissue label‐free proteome and upstream regulator analysis. (A) Enriched proteins in normal PF tissue and Dupuytren's tissue processed without chondroitinase ABC treatment and those in Dupuytren's tissue predicted to be regulated by TGFβ, subdivided based on matrisomal classification. (B) Ingenuity Pathway Analysis (IPA) diagram highlighting matrisomal proteins within those predicted to be regulated by TGFβ in Dupuytren's tissue. Red, increased abundance; green, decreased abundance; orange, predicted to lead to activation; blue, predicted to lead to inhibition; yellow, findings inconsistent with state of downstream molecule; grey, effect not predicted. Matrisomal proteins are indicated with a star (collagens, white; ECM glycoproteins, black; ECM‐affiliated proteins, blue; proteoglycans, red; ECM regulators, yellow; secreted factors, green).

For medium, label‐free analysis and PCA showed normal PF grouped separately from Dupuytren's nodule and cord (supplementary material, Figure S2A). STRING analysis highlighted nine proteins enriched in normal PF, including MMP3 and two well‐connected clusters of matrisomal proteins in Dupuytren's medium (supplementary material, Figure S2B,C). A chondroitinase treatment step was considered for tissue but discounted (Supplementary materials and methods, results). PCA of tissue showed nodule and cord grouped together, though one cord sample overlapped with normal PF (supplementary material, Figure S4A). STRING analysis highlighted 20 proteins in three clusters for normal PF and a well‐connected cluster of matrisomal proteins enriched in Dupuytren's tissue (supplementary material, Figure S4B,C). Reactome pathways are shown in the supplementary material, Table S5. In Dupuytren's medium, 31.0% of enriched proteins were matrisomal, with TGFβ being the top upstream regulator by activation z‐score (supplementary material, Table S6 and Figure S5A). In addition, 46.2% of predicted TGFβ‐regulated proteins were matrisomal (supplementary material, Figure S5B), and 31.4% of proteins enriched in Dupuytren's tissue were matrisomal, with TGFβ again the top potential upstream regulator (supplementary material, Table S7) and 46.3% of the TGFβ‐regulated proteins being matrisomal (Figure 2). Additional pathway analysis results are described in the Supplementary materials and methods. The skewed protein enrichment in Dupuytren's samples, after normalising to tissue wet weight (supplementary material, Figures S2 and S4) indicated proteins may be less extractable from normal PF. Normalisation to total ion chromatogram resulted in a more even distribution between tissue types (supplementary material, Figures S6 and S7). For Reactome pathways, see supplementary material, Table S5. Neopeptides indicative of native proteolytic cleavage were identified in medium and tissue (Supplementary materials and methods, results). For tissue the number of neopeptide sequences was higher for normal PF, and none were restricted solely to Dupuytren's samples, possibly reflecting differences in protein age and specific protease exposure.

There were more newly labelled MMP3 peptides in Dupuytren's than normal PF medium (Figure 1G) but increased abundance in normal PF medium by label‐free analysis (supplementary material, Figures S2A and S6A). Western blotting in a separate set of test samples (Table 1) primarily detected the 57/59 kDa pro‐form [47] of MMP3 in medium and tissue (supplementary material, Figure S8A,B). Quantification relative to total protein (supplementary material, Figure S8C,D) detected no differences in MMP3 in medium or tissue between normal PF and Dupuytren's (supplementary material, Figure S8E,F). Hence, MMP3 turnover may be faster in Dupuytren's tissue than normal PF. Reprobing the membrane verified that fascin (FASCN), identified as more abundant by label‐free proteomics in Dupuytren's tissue by both normalisation methods (supplementary material, Figures S4C and S7B), was more abundant in Dupuytren's tissue extracts (*p* < 0.001) (supplementary material, Figure S8B,G).

### Elevated *COL1A1* mRNA in response to TGFβ isoform treatment particularly in aged rather than young equine tenocytes

**Figure 3:**
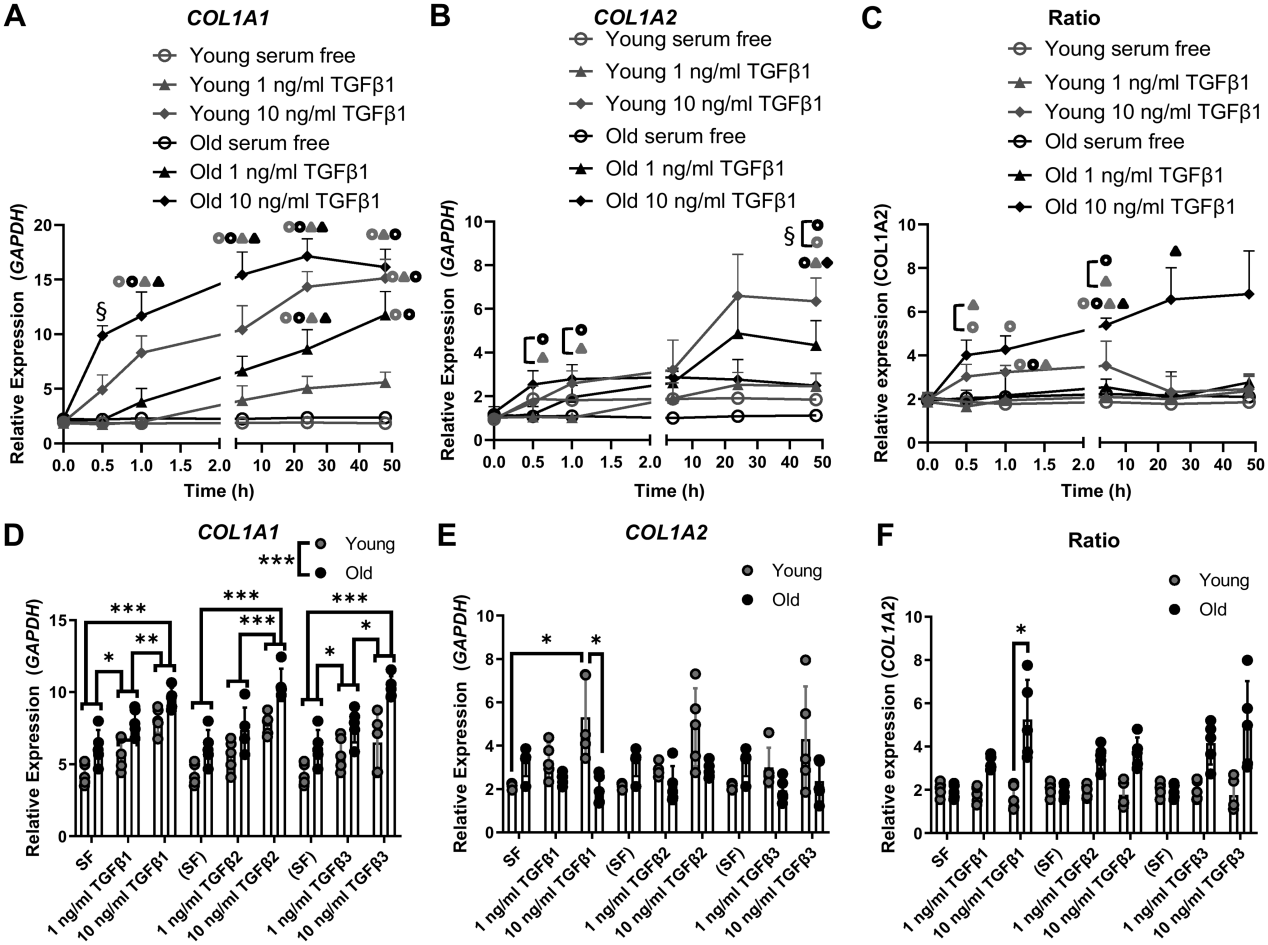
Effect of TGFβ isoforms on type I collagen mRNA expression in young and aged equine tenocytes. (A–C) Time course of mRNA expression for (A) *COL1A1*, (B) *COL1A2*, and (C) *COL1A1:COL1A2* ratio 0, 0.5, 1, 4, 24, and 48 h after control, 1 or 10 ng/ml TGFβ1 treatment. Symbols adjacent to a point indicate conditions with a significant difference to that treatment, symbols with brackets indicate a significant difference where points are too close to indicate such directly, and § indicates significantly different to all other treatments when shown at a time point, or at all time points when symbols are connected with brackets. *n* = 4 (young: 4, 5, 6, and 7 years aged: 15, 17, 22, and 28 years). (D–F) mRNA expression of (D) *COL1A1*, (E) *COL1A2*, and (F) the *COL1A1:COL1A2* ratio 24 h after treatment with control, 1 or 10 ng/ml TGFβ1, TGFβ2, or TGFβ3. The serum‐free (SF) data are shown for each isoform to aid visualisation. *n* = 5 [as for parts A–C plus 4 years (young) and 25 years (aged)]. *p < 0.05, **p < 0.01, and ***p < 0.001. Cross‐isoform comparisons are not indicated. *p* values for all significant comparisons are shown in the supplementary material, Table S4.

To compare TGFβ responsiveness in young and aged cells, equine tenocytes were used as a model system for which both young and aged material could be sourced. The equine lifespan of up to three decades allows for prior adventitious chemical modifications of the ECM that could influence cellular ageing. A time‐course of 10 ng/ml TGFβ1 treatment elevated *COL1A1* mRNA in young and aged equine tenocytes from 24 h (*p* = 0.006 [young], *p* = 0.004 [aged]) to 48 h (*p* = 0.009, *p* = 0.006 respectively) (Figure 3A). By 48 h, *COL1A1* mRNA was higher in aged tenocytes treated with 1 ng/ml TGFβ1 than in cells maintained in serum‐free medium (*p* = 0.05). Conversely, *COL1A2* mRNA by 48 h was highest in young cells treated with 10 ng/ml TGFβ1 and higher in young versus aged tenocytes in serum‐free medium (*p* < 0.001) (Figure 3B). The *COL1A1:COL1A2* mRNA ratio tended to increase in aged cells treated with 10 ng/ml TGFβ1 but not significantly at later time points (Figure 3C). To determine whether different TGFβ isoforms may elicit a different *COL1* mRNA expression response, cells were treated with TGFβ1, TGFβ2, or TGFβ3 for 24 h. Each isoform produced comparable effects, with a dose‐dependent response on *COL1A1* mRNA expression and more pronounced effects in aged cells [*p* < 0.001 (aged versus young)] (Figure 3D). Only TGFβ1 at 10 ng/ml increased *COL1A2* mRNA in young cells (*p* = 0.029 versus serum‐free) (Figure 3E) and elevated the *COL1A1:COL1A2* mRNA ratio in aged cells (*p* = 0.021 versus young) (Figure 3F), while no significant effects were detected for TGFβ2, TGFβ3, or the lower TGFβ1 dose.

### Both *COL1* genes are responsive to TGFβ1 treatment in fibroblasts derived from aged human samples, with higher basal levels in Dupuytren's than in normal PF cells

**Figure 4:**
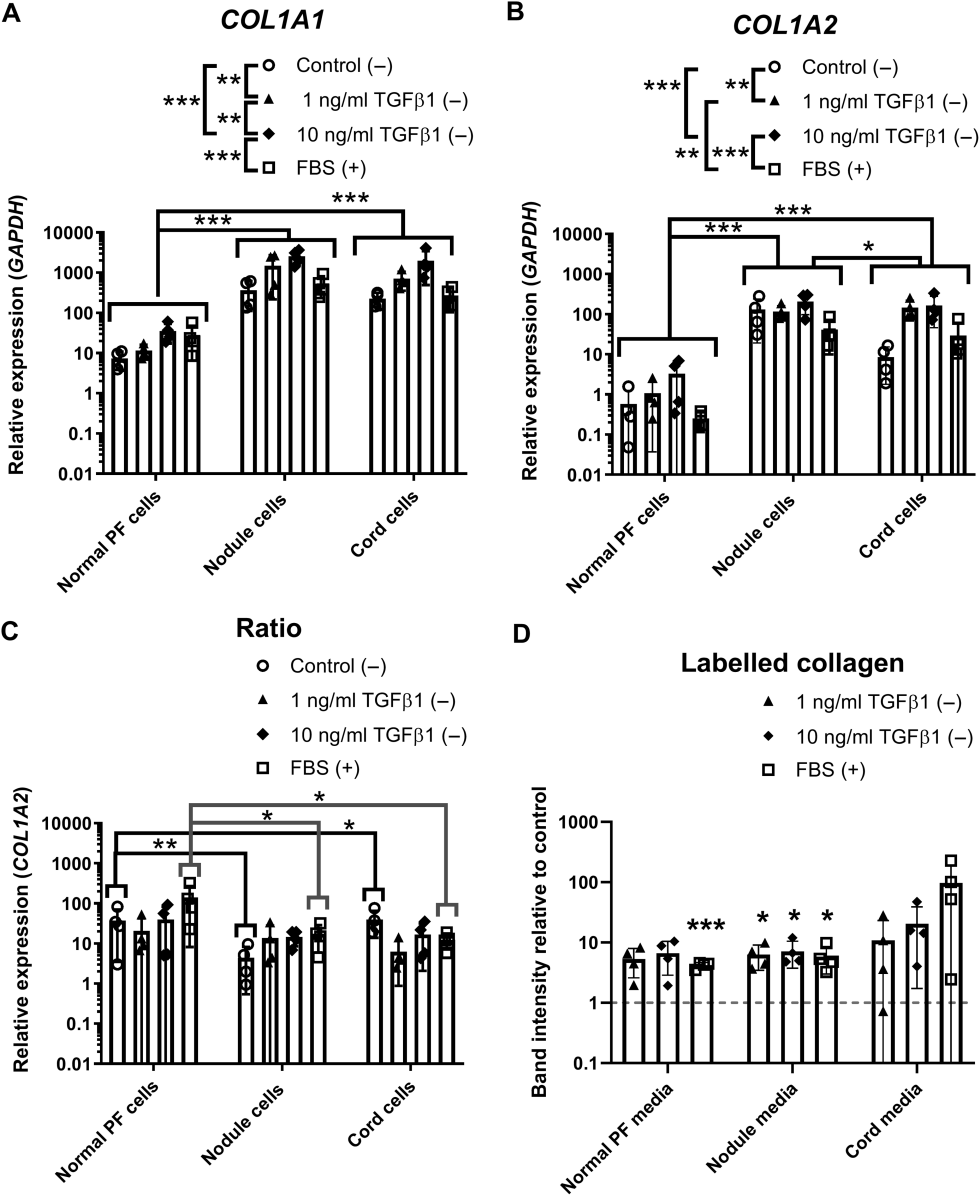
Effect of TGFβ on type I collagen mRNA expression and collagen protein production in Dupuytren's cells. (A–C) Analysis of (A) *COL1A1*, (B) *COL1A2*, and (C) *COL1A1:COL1A2* mRNA expression by RT‐qPCR in normal PF, Dupuytren's nodule, and Dupuytren's cord cells (*n* = 4) after treatment with control, 1 or 10 ng/ml TGFβ treatment in serum‐free conditions (−), or with 10% FBS (+). (D) Densitometric quantification of the relative amounts of radiolabelled (pro)collagen present in conditioned medium from normal PF, Dupuytren's nodule, and Dupuytren's cord cells (*n* = 4) after treatment with 1 or 10 ng/ml TGFβ1 treatment in serum‐free conditions (−), or with 10% FBS (+) compared to serum‐free control. *p < 0.05, **p < 0.01, and ***p < 0.001. *p* values for all significant comparisons are shown in the supplementary material, Table S4. Sample details are given in Table 1.

In aged human cells, *COL1A1* and *COL1A2* mRNA was higher in cells derived from Dupuytren's tissue than normal PF, and both *COL1A1* and *COL1A2* responded to TGFβ1 (*p* < 0.001) (Figure 4A,B), though there were few differences in the *COL1A1:COL1A2* mRNA ratio (Figure 4C). Monitoring of new collagen protein synthesis by analysis of radiolabelled collagen and pro‐forms in the cell culture medium indicated that TGFβ1 increased type I collagen protein in Dupuytren's nodule cell medium [*p* = 0.033 (1 ng/ml), *p* = 0.036 (10 ng/ml)], but not for cord or normal PF (Figure 4D). Pyrosequencing identified no differences in DNA methylation for *COL1A1* or *COL1A2* regulatory regions following TGFβ1 treatment or between cell types (Supplementary materials and methods, results).

### *COL1* genes are not responsive to TGFβ1 in 3D tendon‐like cultures of Dupuytren's and normal PF cells, but cell‐type differences in *COL1A1* mRNA are preserved

**Figure 5:**
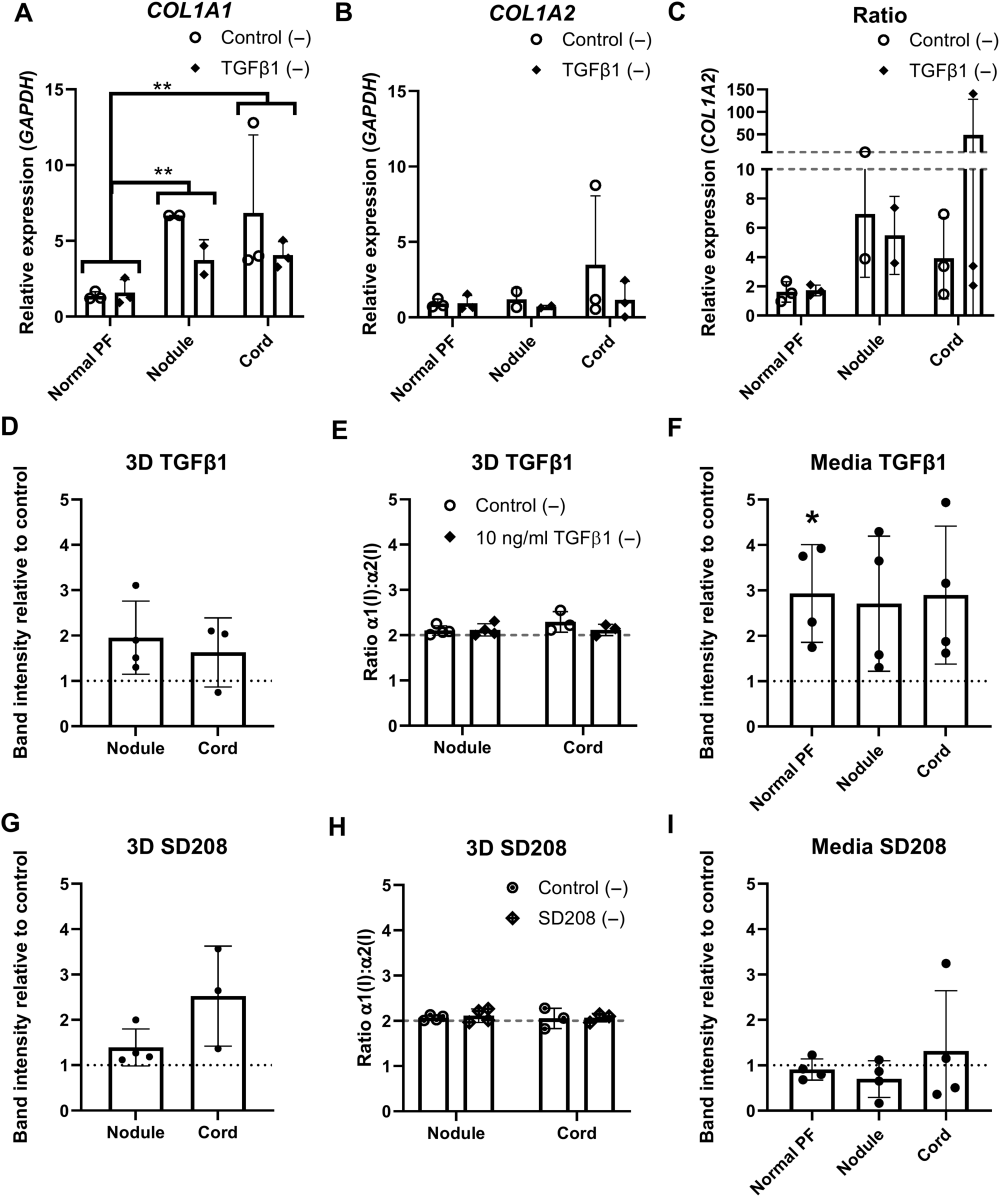
Modulating the TGFβ1 pathway has marginal effects on type I collagen synthesis in 3D tendon‐like constructs derived from normal PF, Dupuytren's nodule, and Dupuytren's cord cells. (A–C) Analysis of (A) *COL1A1*, (B) *COL1A2*, and (C) *COL1A1:COL1A2* mRNA expression by RT‐qPCR in 3D tendon‐like constructs (*n* = 3, except nodule where *n* = 2) after treatment with 10 ng/ml TGFβ1 in serum‐free conditions (−). (D–F) Analysis of (D) type I collagen protein synthesis, (E) type I collagen homotrimer formation, and (F) (pro)collagen secretion by [^14^C]proline labelling in 3D tendon‐like constructs after treatment with 10 ng/ml TGFβ1 in serum‐free conditions (−). (G–I) Analysis of (G) type I collagen protein synthesis, (H) type I collagen homotrimer formation, and (I) (pro)collagen secretion by [^14^C]proline labelling in 3D tendon‐like constructs after treatment with 10 ng/ml TGFβ1 in serum‐free conditions (−) (*n* = 4, except D, E, G, H, where *n* = 4 for nodule and *n* = 3 for cord only). *p < 0.05 and **p < 0.01. Sample details are given in Table 1.

Normal PF and Dupuytren's cells can form 3D tendon‐like constructs that fully process type I collagen and assemble aligned collagen fibrils [18]. Treatment of normal PF or Dupuytren's nodule‐ or cord‐derived tendon‐like constructs with 10 ng/ml TGFβ1 did not alter *COL1A1* or *COL1A2* mRNA expression, or the *COL1A1:COL1A2* ratio (Figure 5A–C), though *COL1A1*, but not *COL1A2*, mRNA was higher in Dupuytren's nodule (*p* = 0.001) or cord (*p* = 0.002) than in normal PF constructs (Figure 5A,B). TGFβ1 treatment did not increase type I collagen protein synthesis (Figure 5D), homotrimer production (Figure 5E), or newly synthesised collagens in cell culture medium (Figure 5F) for Dupuytren's nodule‐ or cord‐derived tendon‐like constructs, though newly synthesised collagen in medium significantly increased after TGFβ1 treatment of normal PF constructs (*p* = 0.037) (Figure 5F). To determine whether TGFβ1 treatment was ineffective in 3D tendon‐like constructs due to persistent TGFβ1 activation, the TGFβ1 inhibitor SD208 was employed. No differences in type I collagen protein synthesis (Figure 5G), homotrimer production (Figure 5H), or newly synthesised collagens in cell culture medium (Figure 5I) were detected after SD208 treatment. However, by two‐way ANOVA, there was a significant difference between the relative amounts of newly synthesised collagens in cell culture medium between TGFβ1 and SD208 treatment across all cell types (*p* < 0.001) (supplementary material, Figure S12).

### Modifying TGFβ signalling does not modulate type I collagen homotrimer production in tissue

**Figure 6:**
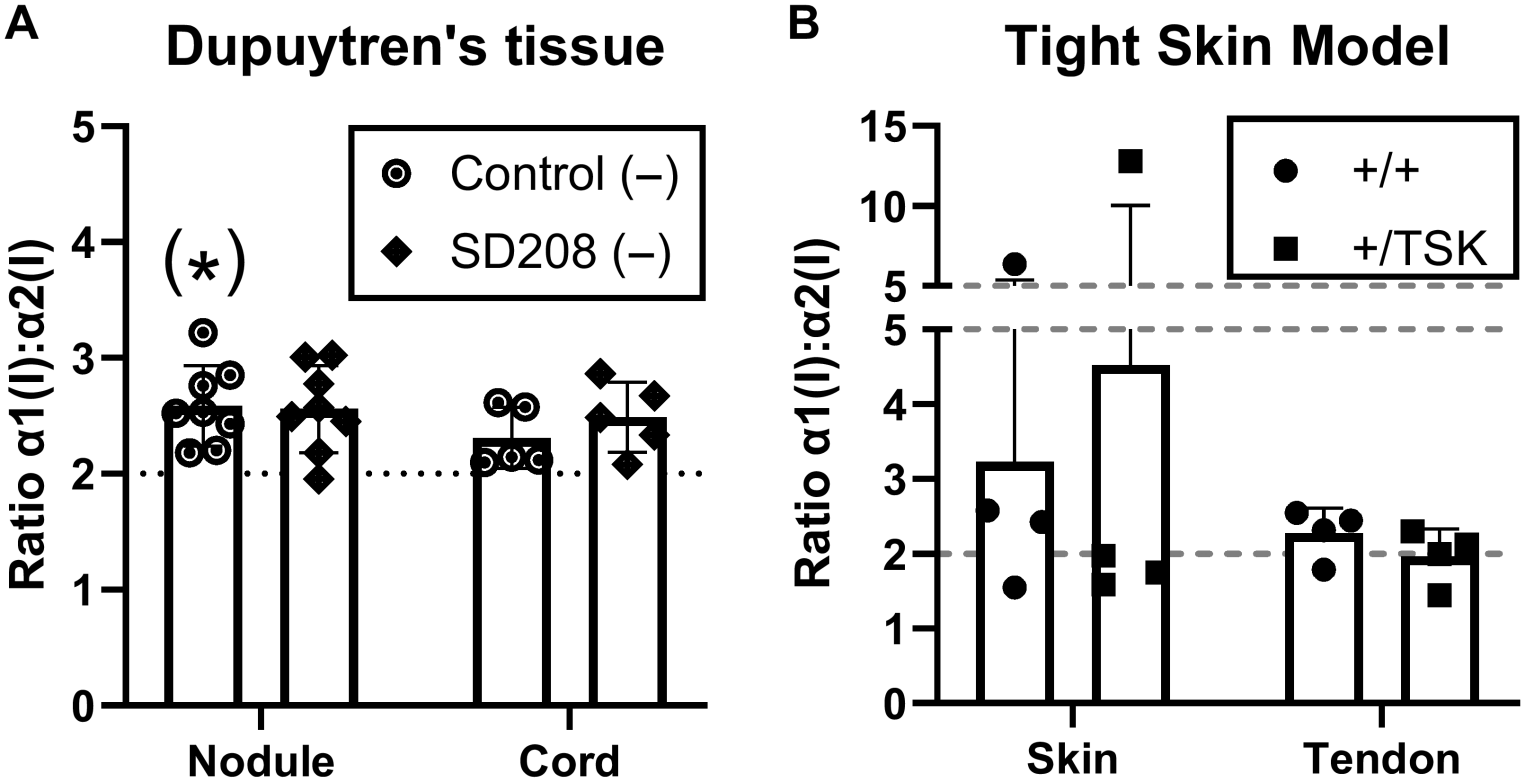
Effect of TGFβ1 pathway modulation in tissue on type I collagen homotrimer synthesis or composition. (A) Analysis of type I collagen homotrimer formation by [^14^C]proline labelling in Dupuytren's nodule (*n* = 8) or cord (*n* = 5) tissue explants after treatment with the TGFβ1 inhibitor SD208, or vehicle control, in serum‐free conditions (−). Samples are detailed in Table 1. (B) Ratio of acid‐soluble type I collagen α1(I) to α2(I) chains as a measure of type I collagen homotrimer content in skin and tendon from the tight‐skin (TSK) mouse (+/TSK heterozygotes) (*n* = 4 per group). *p < 0.05 (1 sample *t*‐tests against a reference value of 2), (*) significant only in a one‐tailed test.

Type I collagen homotrimer production in Dupuytren's tissue explants was not previously abrogated by TNFα treatment [18]. To determine whether TGFβ inhibition influenced type I collagen homotrimer synthesis, Dupuytren's nodule or cord tissue explants were treated with SD208 to block TGFβ signalling. While the α1(I):α2(I) chain ratio tended to be higher than 2, it was not significantly elevated above 2, except in a one‐tailed test for nodule (*p* = 0.002). No differences were observed with TGFβ signalling inhibition with SD208 treatment (Figure 6A). Hence there was only weak evidence for an effect of SD208 on type I collagen homotrimer production in nodule. To evaluate whether altered TGFβ signalling could influence type I collagen homotrimer production, the TSK mouse was used as a model of elevated TGFβ signalling [48, 49]. Acid‐soluble collagen was extracted from WT and TSK heterozygote skin and tail tendon and the α1(I):α2(I) chain ratio determined by SDS PAGE and densitometry. No differences in the ratio were detected between WT and TSK samples or between tissue types by two‐way ANOVA (Figure 6B). Hence, sustained alterations to TGFβ signalling that produce a fibrotic skin phenotype in murine TSK heterozygotes do not produce a corresponding increase in the proportion of acid‐soluble homotrimeric type I collagen in skin or tendon.

## Discussion

This study demonstrated that Dupuytren's tissue actively synthesised matrisomal proteins, with TGFβ1 being a potential regulator of the matrisomal composition of fibrotic tissue. *COL1A1* mRNA was responsive to TGFβ1 in 2D cultured cells from aged equine and human donors with both *COL1A1* and *COL1A2* mRNAs more strongly expressed in Dupuytren's than control cells. Cell type differences in *COL1A1* mRNA were preserved in 3D culture, but, importantly, *COL1* genes were not responsive to TGFβ1 in 3D culture. Interfering with TGFβ signalling in Dupuytren's tissue explants or using the TSK mouse model did not affect type I collagen homotrimer production. Modulating TGFβ pathways in tissues may therefore not directly block excessive collagen deposition in fibrosis.

Metabolic labelling and mass spectrometry confirmed type I collagen synthesis by Dupuytren's samples, as previously demonstrated using radiolabelling [18], with approximately 30% of secreted peptides being labelled. Less than 4% of FN1 peptides were labelled, implying substantial leaching from the tissue. Labelled MMP3 peptides were more abundant in Dupuytren's medium, but MMP3 was more abundant in label‐free analyses of normal PF medium. Western blotting, however, detected no significant differences in MMP abundance, implying faster turnover in Dupuytren's samples. Some leaching of MMP3 degradation products from normal PF tissue could explain the higher MMP3 abundance in normal PF medium in label‐free analyses. Matrix metalloproteinase‐3 (*MMP3*) mRNA expression was previously shown to be lower in Dupuytren's tissue than in normal PF from carpal tunnel patients [20], so post‐transcriptional mechanisms or alterations during explant culture may play a role.

Higher *MMP2* mRNA expression in Dupuytren's tissue correlates with disease recurrence [50] and increased MMP2 activation occurs in Dupuytren's tissue [51]. However, TIMP2 inhibits MMP2, and Dupuytren's tissue has higher tissue inhibitors of metalloproteinases 2 (*TIMP2*) mRNA expression but a lower *MMP2*:*TIMP2* mRNA ratio [52]. Here we found MMP2 and TIMP2 protein to be more abundant and more highly synthesised in Dupuytren's medium. MMP2 facilitates fibroblast‐mediated collagen gel contraction [53], with *MMP2* mRNA being force‐responsive in nodule cells [54]. Hence MMP2 may support contractility, but TIMP2 facilitates ECM deposition via MMP2 inhibition. The MMP2/TIMP2 protein ratio decreases in a murine model of liver fibrosis, an effect ameliorated by IGFBP7 [55]. IGF‐II was previously shown to enhance cell contractility, an effect counteracted by IGFBP6 [56]. IGFBP7 has been implicated in liver fibrosis and is an upstream activator of TGFβ1 [57]. Here, newly synthesised IGFBP7 was present in both Dupuytren's and normal PF media. IGFBP7 modulates IGF signalling and is associated with senescence [58, 59]; therefore, new synthesis may relate to the age of the samples (mean = 64.0 years; range 48–89 years).

TGFβ1 was identified as a likely upstream regulator in Dupuytren's tissue. Immunohistochemistry previously identified each TGFβ isoform in Dupuytren's tissue [26, 27, 52, 60], and each mRNA was identified by RT‐qPCR or *in situ* hybridisation [26, 52, 61, 62]. SMAD pathway activation implicates TGFβ in the disease process [62]; hence, TGFβ1 likely regulates a subset of matrisomal and cellular proteins in Dupuytren's disease. Reactome pathways included those relating to ECM. The normalisation‐dependent association of ECM pathways with normal PF indicates ECM may be more extractable from Dupuytren's tissue, perhaps due to more recent synthesis (as indicated by the labelling results) and reduced protein crosslinking. Active ECM synthesis and turnover in Dupuytren's samples is consistent with the fibroproliferative disease process.

*COL1A1* and *COL1A2* mRNA expression generally decreases with age, but aged fibroblasts can be more prone to pro‐fibrotic induction *in situ* [63]. Higher *COL1A1* and *COL1A2* mRNA in 2D cultured Dupuytren's than normal PF cells matches previous observations and suggests the cells retain tissue‐of‐origin characteristics as for the myofibroblast phenotype [64]. TGFβ1 treatment increased both *COL1A1* and *COL1A2* mRNAs across human cell types, in line with previous findings for *COL1A1* [25]. No alterations in *COL1A1* or *COL1A2* DNA occurred after TGFβ1 treatment, unlike in neonatal rat cardiac fibroblasts in which *COL1A1* promoter regions became demethylated [65]. DNA methylation levels were, however, very low, possibly in connection with age‐related global DNA hypomethylation [66]. Increased collagen protein synthesis was only detected in media of nodular cells following TGFβ1 treatment, though this previously occurred in normal PF and Dupuytren's cells for cells approximately two decades younger [67]. Age‐related changes in post‐transcriptional regulation and translation of *COL1* mRNAs [68] could affect whether mRNAs transcribed in response to TGFβ1 treatment result in additional type I collagen protein synthesis.

In 3D cultures, cell type differences in *COL1A1* mRNA expression were preserved. TGFβ and SD208 had opposite effects on new collagen detected in medium, although the lack of more pronounced effects may relate to treating cells embedded in a dense cell‐derived collagenous ECM. Other potential factors may include negative feedback with high‐dose TGFβ [69], a chronically active myofibroblast phenotype being refractory to further TGFβ stimulation [70], differences in ECM stiffness, or altered ECM‐receptor signalling. There was no evidence of elevated homotrimer synthesis in 3D, or its induction by TGFβ1, consistent with findings in 2D primary human lung fibroblasts [71] and cancer‐associated fibroblasts [72]. In tissue, the TGFβ signalling inhibitor SD208 did not significantly alter type I collagen homotrimer synthesis, though in this sample set, unlike previously [18], there was limited evidence for new homotrimer synthesis. TGFβ pathway activation in Dupuytren's disease may result from altered TGFβ1 release and degradation kinetics, as least in 2D [73].

Here the TSK mouse was harnessed as an alternative approach to modulating TGFβ signalling in tissue. TSK has a multiple exon duplication in fibrillin‐1, a component of microfibrils in elastic fibres, and develops skin fibrosis [49]. Microfibrils contain latent TGFβ binding proteins, and fibrillin‐1 mutations in human Marfan syndrome can be counteracted by TGFβ inhibition. While TSK has over‐active TGFβ‐signalling, it did not have an increased proportion of type I collagen homotrimer. Hence TGFβ does not promote type I collagen homotrimer synthesis in tissue.

Therapeutic targeting of TGFβ is possible, with clinical trials ongoing, primarily in oncology applications [29], and localised injection into Dupuytren's tissue may circumvent some of the safety concerns. Indeed, pirfenidone has shown promise in preclinical studies for modulating TGFβ and is in development as an injectable [74]. Several other cytokines and signalling pathways have been implicated in Dupuytren's disease, and an unbiased screening approach could help identify the most effective approach to blocking collagen production in fibrosis.

## Author contributions statement

The study was conceived by EGC‐L. Funding was acquired by PDC and EGC‐L. Methodology was developed by GC, JAG, KJL, TL and EGC‐L. Study investigations were undertaken by GC, JAG, SA, KJL, AC, HC and RI. Data curation was performed by GC, SA, KJL, AC, HC and EGC‐L. Project administration was conducted by PDC and EGC‐L. Study supervision was provided by EB, TL, PDC and EGC‐L. Formal analysis was conducted by GC, SA and EGC‐L. Visualisation was performed by GC, JAG, SA, NSO‐Y, LAM and EGC‐L. NSO‐Y and EGC‐L wrote the original draft. Writing approval was provided by GC, SA, KJL, AC, HC, NSO‐Y, RI and LAM. The manuscript was reviewed and edited by JAG, EB, TL, PDC and EGC‐L.

## Supporting information


Supplementary materials and methods
**Figure S1.** Experimental design for proteomics, and cell and tissue treatments
**Figure S2.** Label‐free proteomics analysis of normal PF and Dupuytren's explant media
**Figure S3.** Evidence for loss of proteins to supernatant with chondroitinase ABC treatment
**Figure S4.** Label‐free proteomics analysis of normal PF and Dupuytren's explant tissue processed without chondroitinase ABC treatment
**Figure S5.** Matrisomal enrichment in normal PF and Dupuytren's explant media and upstream regulator analysis
**Figure S6.** STRING interaction networks for normal PF and Dupuytren's explant proteomes from medium normalised using total ion chromatogram
**Figure S7.** STRING interaction networks for normal PF and Dupuytren's explant proteomes from tissue normalised using total ion chromatogram
**Figure S8.** Western blotting analysis of selected proteins identified using proteomics
**Figure S9.** Locations of regions analysed by pyrosequencing in *COL1* genes
**Figure S10.** TGFβ treatment does not affect CpG methylation in human *COL1* regulatory regions
**Figure S11.** Methylation levels at individual CpG sites in human *COL1* regulatory regions with TGFβ1 treatment
**Figure S12.** Comparison of amount of labelled collagen in medium of tendon‐like constructs treated with TGFβ1 or inhibitor SD208
**Table S1.** Primer sequences used for RT‐qPCR
**Table S2.** Primer sequences for pyrosequencing analysis
**Table S3.** Location of analysed *COL1* CpG sites on chromosomal assemblies and transcripts
**Table S4.** Table of *p* values and statistical tests
**Table S5.** Reactome pathways from STRING
**Table S6.** Top 10 upstream regulators identified using IPA for media
**Table S7.** Top 10 upstream regulators identified using IPA for tissue
**Table S8.** Top 10 diseases and bio‐functions identified using IPA for media
**Table S9.** Top 10 diseases and bio‐functions identified using IPA for tissue
**Table S10.** Number of unique neopeptide sequences in each sample type filtered for occurrence in at least three samples of normal PF, Dupuytren's nodule, or cord media samples
**Table S11.** Number of unique neopeptide sequences in each sample type filtered for occurrence in at least three samples of normal PF, Dupuytren's nodule, or cord tissue samples

## Data Availability

The mass spectrometry proteomics data have been deposited at the ProteomeXchange Consortium via the PRIDE partner repository (http://www.ebi.ac.uk/pride/archive/) with the data set identifier PXD069194. The data presented in this study are openly available in DataCat at https://doi.org/10.17638/datacat.liverpool.ac.uk/3056.
