# Supplementary material for: Uncoupling TGFβ1 signalling from collagen protein synthesis in Dupuytren's disease
Source: J Pathol. 2026 Jan 26;268(4):383–97. doi: 10.1002/path.70020 (PMC12984003; doi:10.1002/path.70020)
Supplement: Supplementary file 1 — Supplementary materials and methods Figure S1. Experimental design for proteomics, and cell and tissue treatments Figure S2. Label‐free proteomics analysis of normal PF and Dupuytren's explant media Figure S3. Evidence for loss of proteins to supernatant with chondroitinase ABC treatment Figure S4. Label‐free proteomics analysis of normal PF and Dupuytren's explant tissue processed without chondroitinase ABC treatment Figure S5. Matrisomal enrichment in normal PF and Dupuytren's explant media and upstream regulator analysis Figure S6. STRING interaction networks for normal PF and Dupuytren's explant proteomes from medium normalised using total ion chromatogram Figure S7. STRING interaction networks for normal PF and Dupuytren's explant proteomes from tissue normalised using total ion chromatogram Figure S8. Western blotting analysis of selected proteins identified using proteomics Figure S9. Locations of regions analysed by pyrosequencing in COL1 genes Figure S10. TGFβ treatment does not affect CpG methylation in human COL1 regulatory regions Figure S11. Methylation levels at individual CpG sites in human COL1 regulatory regions with TGFβ1 treatment Figure S12. Comparison of amount of labelled collagen in medium of tendon‐like constructs treated with TGFβ1 or inhibitor SD208 Table S1. Primer sequences used for RT‐qPCR Table S2. Primer sequences for pyrosequencing analysis Table S3. Location of analysed COL1 CpG sites on chromosomal assemblies and transcripts Table S4. Table of p values and statistical tests Table S5. Reactome pathways from STRING Table S6. Top 10 upstream regulators identified using IPA for media Table S7. Top 10 upstream regulators identified using IPA for tissue Table S8. Top 10 diseases and bio‐functions identified using IPA for media Table S9. Top 10 diseases and bio‐functions identified using IPA for tissue Table S10. Number of unique neopeptide sequences in each sample type filtered for occurrence in at least three samples of normal PF, Dupuyt [file PATH-268-383-s001.docx]

# Uncoupling TGFβ1 signalling from collagen protein synthesis in Dupuytren’s disease

#

G Cooper *et al. J Pathol* <https://doi.org/10.1002/path.70020>

**Supplementary materials and methods**

**Supplementary results**

**Supplementary Figures S1–S12**

**Supplementary Tables S1–S11**

Reference numbers refer to the main text list

**Supplementary materials and methods**

## **Sample preparation for mass spectrometry**

Tissue samples were disrupted using a Mikro-Dismembrator (Braun International, Melsungen, Hessen, Germany) under liquid nitrogen. Approximately 10 mg of tissue was weighed directly in LoBind tubes (Eppendorf, Hamburg, Germany), snap frozen and stored at −80 °C. For prior chondroitinase treatment, 80 μl of chondroitinase ABC (AMSBIO, Abingdon, Oxfordshire, UK) (AMS.E1028-10) at 1 U/ml in 100 mM Tris Acetate (Sigma-Aldrich, St. Louis, MO, USA) containing protease inhibitors with EDTA (complete protease inhibitor cocktail, Roche, Basel, Switzerland) was used per 10 mg of homogenised tissue and the chondroitinase supernatant reserved (*n* = 4 except nodule for which *n* = 3). A sequential GnHCl followed by 0.1% RapiGest (Waters, Milford, MA, USA) extraction was carried out as described elsewhere [75], except that 100 μl of GnHCl extraction buffer containing 1 mM EDTA (Sigma Aldrich) was used per 10 mg of starting material, the subsequent steps scaled similarly, and the final Rapigest pellet extraction was not carried out. For tissue extracts and chondroitinase supernatants, filter-aided sample preparation (FASP) was carried out with centrifugation steps at 12,500 rpm for 15 min, unless otherwise indicated. 100 μl of formic acid (1% v/v) was added to each filter (Vivacon 500, 10,000 MWCO, Sartorius, Göttingen, Lower Saxony, Germany) and spun. Extract from an equivalent of 2 mg of tissue or the entire chondroitinase supernatant was added to each filter and made up to 200 μl with 4 M GnHCl (Sigma Aldrich) in 50-mM ammonium bicarbonate (Sigma Aldrich) (GnHCl buffer). A 15-min incubation in 8 mM DTT (Sigma Aldrich) in GnHCl buffer at 56 ^o^C in the dark with gentle vortexing preceded a 10-min spin. Each filter was washed twice with 100 μl 4 M GnHCl buffer and spun. 100 μl of 50 mM iodoacetamide (Sigma Aldrich) in GnHCl buffer was used to alkylate proteins with gentle vortexing and a 20-min incubation in the dark before a 10-min spin. Each filter was washed twice with 100 µl GnHCl buffer, then three times with 50 mM ammonium bicarbonate with a 10-min spin. Bound proteins were digested with 40 μl trypsin (Sigma Aldrich) at 10 ng/μl in 47.5 mM ammonium bicarbonate with 2.5 mM acetic acid (Sigma Aldrich) at 37 °C overnight with steps to prevent evaporation, followed by a 10-min spin and a subsequent wash with 40 μl of 50 mM ammonium bicarbonate. Combined flow-throughs were acidified with trifluoroacetic acid (VWR International, Radnor, PA, USA) to 0.2% (v/v). Incubation medium was bound to and digested off Strataclean beads (Agilent Technologies, Santa Clara, CA, USA), as previously described [75,76]. A yeast enolase peptide standard (Waters) was added to each sample, though this was not utilised for later quantification.

**Proteomic data analysis**

Protein identification, label-free quantification, and analysis of ^13^C_6_-L-lysine labelling were carried out as previously described [37], with the following modifications. *De novo* and database PEAKS searches using Peaks Studio version 8 (Bioinformatics Solutions, Waterloo, Ontario, Canada) were carried out using the UniHuman Database with Waters Pepmix. Fragment mass error tolerance was set to 0.02 Da in searches, and proteins were filtered using −10lgP ≥ 20 threshold. Results were normalised to the equivalent wet weight of tissue loaded on the trapping column or using the total ion chromatogram (TIC) and analysed using PeaksQ (Peaks Studio version 8). The Dupuytren’s:Normal PF ratio for each protein was calculated from group profile ratios as (Nodule+Cord)/(Normal PF × 2), converted to an expression fold-change in IPA (Ingenuity Pathway Analysis, Qiagen, Hilden, Germany), and used for core expression analysis. The Dupuytren’s:Normal PF ratio was used to define proteins associated with each tissue type, for which protein interactions and pathways were analysed using STRING version 11.0 [77] using default settings, except that ‘molecular action’ was used to define network edges, and k-means clustering with five or three clusters was used for proteins enriched in Dupuytren’s or normal PF tissue respectively. Samples were allocated for the proteomics workflow prior to final confirmation of patient age and gender information on the day of surgery. Datasets were subsequently selected for inclusion in the analysis to minimise key differences between groups and facilitate statistical analysis.

Neopeptide analysis was carried out as previously described [78], with the following modifications. The UniHuman Database (https://www.uniprot.org/proteomes/UP000005640) was used via a local Mascot server (Matrix Science, London, UK), and search parameters were enzyme, semiTrypsin; fragment mass tolerance; 0.01 Da; peptide charge; 2+; monoisotopic, instrument, ESI-QUAD-TOF; and decoy search selected. Peptide modifications were fixed carbamidomethyl cysteine, variable oxidation of methionine, and variable heavy [^13^C(_6_)] lysine. Results were filtered to include only those neopeptides present in at least three samples of normal PF, Dupuytren’s, nodule, or cord samples, without editing for overlapping or similar peptides in the same or other samples.

**Western blotting**

Normal PF and Dupuytren’s tissue extracts and media used for western blotting (Table 1) were derived from explants labelled with 2.5 μCi/ml [^14^C]proline (Perkin Elmer, Shelton, CT, USA) overnight without cytokine or serum treatment, or incubated in unlabelled media, before extraction with salt extraction buffer [18]. A further salt/NP40 extraction was subsequently carried out to extract intracellular proteins [79]. Tissue extracts and conditioned media from incubated tissue explants were stored at −20 °C before analysis. For tissue extracts, equal volumes of salt extracts and salt/NP40 extractions were pooled before analysis. Tissue extracts or media from separate cord and nodule Dupuytren’s samples were also pooled for western blotting. Samples were analysed by electrophoresis on 10% pre-cast Tris-Glycine mini protein gels (Thermo Fisher Scientific, Waltham, MA, USA) and proteins transferred to 0.2 µm nitrocellulose membranes (Thermo Fisher Scientific) before staining with Ponceau S (0.1% in 5% acetic acid) (Sigma Aldrich). Membranes were blocked overnight in 5% non-fat dried milk powder (Tesco, Welwyn Garden City, Hertfordshire, UK) in PBS (Thermo Fisher Scientific) with 1% Tween 20 (Sigma Aldrich) (PBS-T). Washes were carried out once for 15 min and three times for 5 min with PBS-T before and after each antibody incubation for 1 h at room temperature in 2% non-fat dried milk powder in PBS-T. The primary antibodies used were rabbit anti-MMP3 (17873-1-AP, lot number 00015874, Proteintech, Rosemont, IL, USA) and rabbit anti-fascin (14384-1-AP, lot number 00058645, Proteintech). The secondary antibody was mouse anti-rabbit IgG-HRP (sc-2357, Santa Cruz Biotechnology, Dallas, TX, USA) at 0.04 µg/ml. Chemifluorescence detection was performed using Pierce ECL Plus Western Blotting Substrate (Thermo Fisher Scientific), and a Typhoon FLA7000 IP imager (GE Healthcare, Chicago IL, USA) with Cy2 filter. Densitometry was used with ImageQuant (GE Healthcare) and a ‘rolling ball’ background. The regions quantified in each lane corresponded to those of the visible bands for MMP3 and fascin, while the whole lane was considered for total protein. The band intensity corrected for total protein detected in each lane was then used to calculate a corrected band intensity for MMP3 and fascin.

**RT-qPCR detailed method**

For 2D cultured cells, RNA extraction, purification, and cDNA synthesis were carried out as previously described [39]. RNA was extracted from all cell types by applying Trizol (Thermo Fisher Scientific) to the cell monolayers and using a cell scraper to detach the cells. After vortexing and centrifugation, 50 μg/ml GlycoBlue (Thermo Fisher Scientific) and 100% isopropanol (Sigma Aldrich) were added to the aqueous phase for RNA precipitation. Following centrifugation, the RNA pellets were washed in 75% ethanol (Sigma Aldrich) and resuspended in Tris-EDTA buffer (Thermo Fisher Scientific). The quantity and quality of RNA was assessed using a NanoDrop spectrophotometer (Thermo Fisher Scientific). DNase (4 U) (Thermo Fisher Scientific) was added to the samples to remove DNA, after which an equal volume of phenol:chloroform:IAA (Thermo Fisher Scientific) was added to each sample. RNA was precipitated, centrifuged, and washed in ethanol and the RNA quality assessed. cDNA was synthesised in a 25-μl reaction from 1–2 μg of total RNA. The conditions for cDNA synthesis were incubation for 5 min at 70 °C, 60 min at 37 °C, and 5 min at 93 °C with M-MLV reverse transcriptase and random-hexamer oligonucleotides (Promega, Madison, WI, USA). RT-qPCR was then performed using Takyon MasterMix Plus, ROX for SYBR Assay (Eurogentec, Liege, Belgium) on a AB7300 instrument (Applied Biosystems, Waltham, MA, USA) with 4 ng/µl cDNA template per reaction.

Three-dimensional tendon-like constructs were immersed in RNAlater (Qiagen) and stored at −20 °C until analysis. RNA extraction and qPCR were performed for 3D constructs as previously described [18]. Constructs were homogenised using a steel ball lysing matrix and a FastPrep 24 tissue homogeniser (MP Biomedicals, Santa Ana, CA, USA). RNA was extracted from homogenised samples using an RNeasy kit (Qiagen) following the manufacturer’s protocol. cDNA was synthesised in a 25-µl reaction from 0.15 µg for constructs as described. cDNA synthesis and qPCR were performed as previously reported [80]. The conditions for cDNA synthesis were as follows: incubation for 5 min at 70 °C, 60 min at 37 °C, and 5 min at 93 °C in the presence of 1 U/µl RNasin ribonuclease inhibitor (Promega), 2 mM PCR nucleotide mix (Promega), 8 U/µl M-MLV reverse transcriptase (Promega), and 0.02 µg/µl random-hexamer oligonucleotides (Promega) per reaction. RT-qPCR was conducted using a Takyon ROX Master Mix containing SYBR Green DNA intercalating dye (Eurogentec). In a 20-µl reaction, 10 ng cDNA was amplified in a Lightcycler 96 qPCR machine (Roche). After a Takyon activation for 3 min at 95 °C, 40 PCR cycles were performed consisting of 10 s at 95 °C and 45 s at 60 °C.

Primer (Eurogentec) sequences are listed in the supplementary material, Table S1. Primer-BLAST (<https://www.ncbi.nlm.nih.gov/tools/primer-blast/>, date last accessed 3 December 2025) was used for primer design and the quality of each primer tested using NetPrimer (<https://www.premierbiosoft.com/netprimer/>, date last accessed 3 December 2025). Each primer was also subjected to a BLAST (<https://blast.ncbi.nlm.nih.gov/Blast.cgi>, date last accessed 3 December 2025) search to confirm specificity. Single amplicons were verified by melt-curve analysis and primer efficiencies verified to lie between 90 and 110%. *GAPDH* was determined to be a suitable reference gene after assessing its stability using the geNorm method [81]. Expression levels of mRNA were calculated relative to *GAPDH* (or to *COL1A2* when determining the *COL1A1:COL1A2* mRNA ratio) using the 2^−ΔCt^ method [40].

**Pyrosequencing**

For pyrosequencing, cells were dissociated using 0.05% trypsin (Thermo Fisher Scientific) and then centrifuged for 5 min at 300 × *g*. The pellet was re-suspended in 200 µl PBS, and 20 µl of proteinase K (Qiagen) was added. DNA was extracted from Carpal ligament and Dupuytren’s fibroblasts using a Dneasy Blood & Tissue Kit (Qiagen), following the manufacturer’s protocol. DNA quantity and quality were evaluated by 240/260/280 nm spectroscopy using a NanoDrop spectrophotometer (Thermo Fisher Scientific).

A methylation standard curve made from human lymphocyte DNA obtained from the Liverpool Lung Project Biobank (https://liverpoollungproject.org.uk/biobank-2/, date last accessed 3 December 2025), which was methylated *in vitro* using SssI methylase (New England Biolabs, Ipswich, MA, USA) following the manufacturer’s protocol. Methylated DNA was mixed in differing ratios with unmethylated lymphocyte DNA to generate standards: 0, 20, 40, 60, 80, and 100% methylated DNA. 500 ng–1 µg of isolated (sample) DNA and DNA standards were bisulphite converted using the EZ DNA Methylation-Gold^TM^ Kit (Zymo Research, Irvine, CA, USA) following the manufacturer’s protocol.

PCR was performed in a 25 µl final volume comprising HotStarTaq Plus polymerase (Qiagen; 0.13 µl), 10× HotStarTaq PCR buffer (2.5 µl), 20 mM dNTP stock (5 mM each dNTP) (Promega; 1 µl), primer mix (1 µl; 3.5 µM biotinylated, 7 µM non-biotinylated), 2 µl bisulphite converted DNA template, and 18.4 µl water. Primer (Eurofins Genomics, Wolverhampton, UK) sequences are provided in the supplementary material, Table S2. The locations of the CpG sites relative to the chromosomal assemblies and transcripts are shown in the supplementary material, Table S3. PCR was optimised for annealing temperature and annealing/extension time to guarantee a single band on a 2% agarose gel, free of artefacts. The final PCR cycling conditions were 95 °C for 5 min, followed by 40 cycles of 94 °C for 15 s to denature the DNA, 50–60 °C for 30 s (annealing), and 72 °C for 5 s (extension), followed by 72 °C for 10 min (final extension).

The PCR product was mixed with 75 μl binding mix (23 μl deionised water, 2 μl streptavidin-coated sepharose beads (GE Healthcare) and 50 μl PyroMark binding buffer (Qiagen) for 20 min. A Pyromark Q24 Vacuum Workstation (Qiagen) was used to prepare the plate for the pyrosequencer following the manufacturer’s protocol. The sepharose beads were captured with filter probes, followed by 10 s cleanup steps with 70% ethanol, 0.2 M sodium hydroxide (Sigma Aldrich), and 100 mM Tris acetate and then mixed with 43.5 μl PyroMark annealing buffer (Qiagen) and 1.5 μl sequencing primer per well. The annealing plate was denatured at 80 °C for 2 min, followed by annealing at room temperature for 2 min. Pyrosequencing was performed using a Pyromark 96MD instrument (Qiagen). Two PCR replicates per sample were analysed by pyrosequencing.

## **Supplementary results**

**Evaluation of chondroitinase ABC pre-treatment of tissue for mass spectrometry**

Our previous proteomic analysis of tendon and ligament tissue included a chondroitinase ABC treatment step to remove highly negatively charged glycosaminoglycans [82], as did earlier studies [78,83] and the work on which we based our guanidine/RapiGest extraction methodology [75]. Removal of large negatively charged glycosaminoglycans could improve proteoglycan identification by improving protein digestion and peptide fractionation, but chondroitinase digestion also generates a supernatant to which some proteins could be lost. During tissue preparation in the present study, the chondroitinase ABC treatment step was either omitted or included and the chondroitinase supernatant reserved, and protein abundance was compared between untreated, treated, and supernatant. Heatmaps indicated some loss of proteins to the chondroitinase ABC supernatant, with increased abundance of several proteins in the supernatant of normal PF and Dupuytren’s cord samples (supplementary material, Figure S3). While arguably chondroitinase ABC treatment increased the detection of some proteins in Dupuytren’s nodule and cord, it generally decreased detection in normal PF, and approximately equal numbers of proteins showed decreased versus increased abundance with chondroitinase treatment in Dupuytren’s cord. For normal PF and cord there was evidence of some loss of proteins to the chondroitinase supernatant. Analysis was therefore performed on samples without the chondroitinase treatment step. There was no overt difference in the appearance of the PCA plot for untreated and chondroitinase-treated samples (not shown).

**Pathway analysis**

The top 10 upstream regulators by activation z-score, filtered for genes, RNAs, and proteins, are listed in the supplementary material, Table S6 for media and Table S7 for tissue. Canonical pathways only had a small number of proteins associated with each pathway and are not reported. The top 10 associated diseases and biofunctions are listed in the supplementary material, Table S8 for media and Table S9 for tissue. Both media and tissue had top-scoring biofunctions associated with cellular movement, cellular compromise, organismal injury and abnormalities, inflammatory responses, and skeletal/muscular disorders.

**Neopeptide analysis**

Neopeptides were identified and filtered by occurrence in at least three samples of normal PF, Dupuytren’s nodule, or cord medium (supplementary material, Table S10) or tissue (supplementary material, Table S11). Neopeptides for proteoglycan (PRG4) were present solely in normal PF medium for at least three samples, while those for adipocyte enhancer-binding protein (AEBP1), collagen alpha-1(IV) chain (COL4A1), and peptidyl-prolyl cis-trans isomerase FKBP1A (FKBP1A) were present solely in Dupuytren’s medium with the same filter (supplementary material, Table S10). For tissue, neopeptides for cartilage intermediate layer protein 1 (CILP), fibulin-1 (FBLN1), and vitronectin (VTN) were present solely in at least three normal PF samples, while no neopeptides were restricted only to at least three Dupuytren’s nodule or cord samples (supplementary material, Table S11).

None of the type I collagen neopeptides corresponded to the MMP cleavage site. The collagen alpha-2(I) chain peptide PGPMGLMGPR identified in all five normal PF but no Dupuytren’s samples corresponds to the start of the triple-helical region, indicating potential loss of the N-telopeptide. The collagen alpha-1(VI) chain peptide YAELLEDAFLK is located at the start of the second von Willebrand factor domain of the NC1 domain, indicating potential proteolytic release. The decorin IVIELGTNPLK peptide, identified in all five normal PF but no Dupuytren’s samples, is located within the fifth of 10 leucine-rich repeats, while the fibromodulin neopeptide CTVVDVVNFSK is at the end of the 10th and final leucine-rich repeat. The vitronectin neopeptide SIAQYWLGCPAPGH is at the C-terminal end of the molecule but lacks the C-terminal leucine.

**DNA methylation analysis for regulatory regions of type I collagen genes**

TGFβ1 has been reported to act via demethylation of the *COL1A1* promoter in neonatal rat cardiac fibroblasts [65]. To determine whether TGFβ1 could act by altering *COL1* DNA methylation in Dupuytren’s cells, CpG-rich regions predicted to be hypermethylated in previous studies [84] or identified with the EMBOSS Cpgplot online tool (<https://www.ebi.ac.uk/jdispatcher/seqstats/emboss_cpgplot>, date last accessed 3 December 2025) were analysed (supplementary material, Figure S9). The analysis also included an Sp1 binding site region in the first intron of *COL1A1* that includes a single-nucleotide polymorphism (‘s’ allele, +1245G/T, rs1800012) that influences *COL1A1* mRNA expression [85] and is adjacent to a CpG site (supplementary material, Table S3). No differences in DNA methylation were identified in any region following TGFβ1 treatment or between cell types (supplementary material, Figures S10 and S11). Only the *COL1A1* −1500 region had methylation levels over 10% (supplementary material, Figures S10B and S11B), whereas the *LINE1* element (an indicator of global genome methylation) had methylation levels over 50% (supplementary material, Figures S10G and S11G).


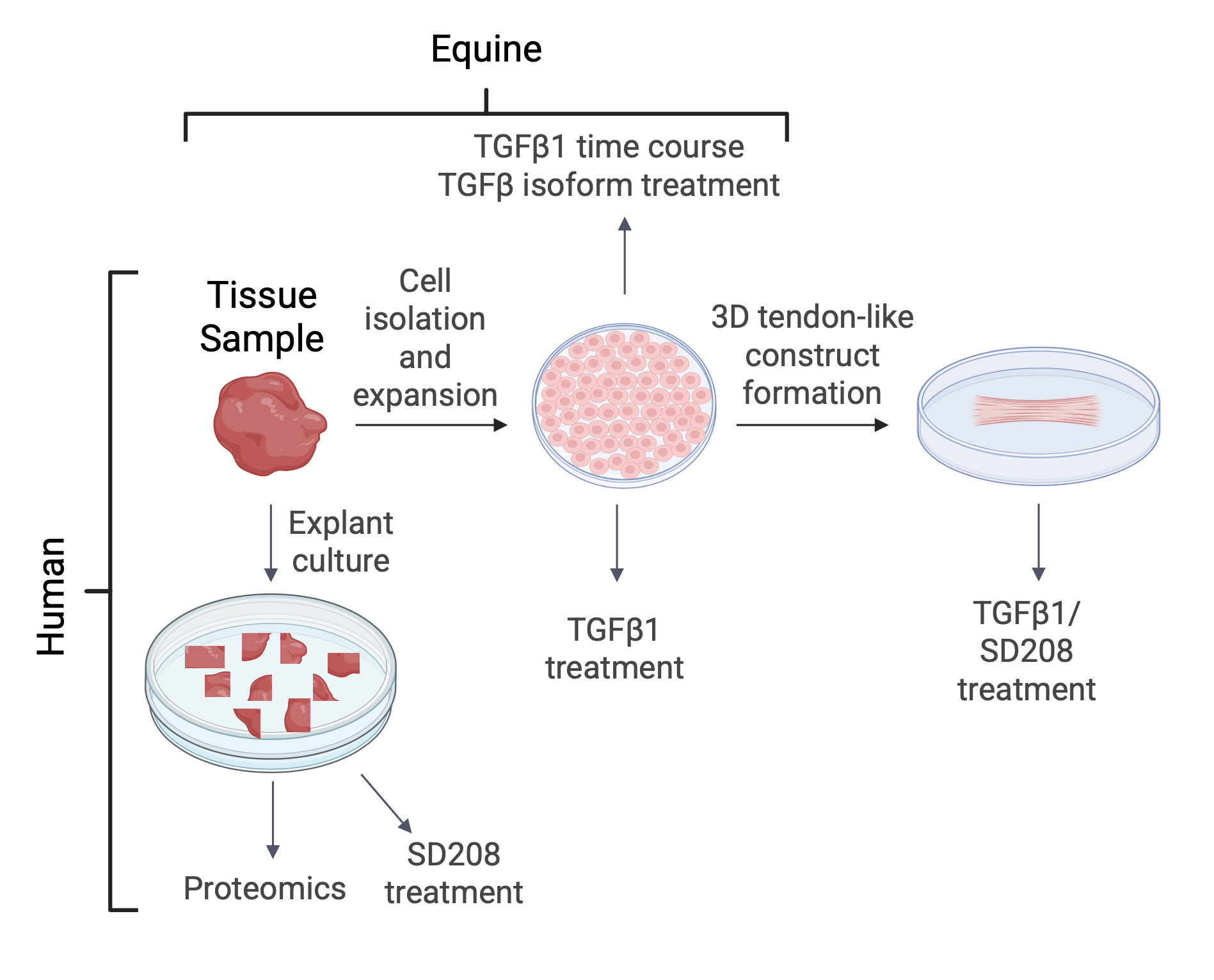


**Figure S1. Experimental design for proteomics and for cell and tissue treatments.** Human samples were used for explant culture, TGFβ1 treatment of 2D cultures, and both TGFβ1 and SD208 treatment of 3D tendon-like constructs. Equine tenocytes were used for TGFβ1 time-course and TGF isoform treatments. Created in BioRender. Laird, E. (2026) <https://BioRender.com/qqxv05o>





**Figure S2. Label-free proteomics analysis of normal PF and Dupuytren’s explant media.** (A) PCA score plot grouped by tissue type. (B) STRING interaction network for proteins enriched in normal PF explant media. (C) STRING interaction network for proteins enriched in Dupuytren’s explant media. Circle colours indicate k-means clusters of proteins. Line colours indicate activation (green), binding (blue), phenotype (cyan), reaction (black), inhibition (red), catalysis (purple), post-translational modification (pink), or transcriptional regulation (yellow). Line ends indicate positive (arrowhead), negative (orthogonal line), or unspecified effect (circle). Matrisomal proteins are indicated by a star (collagens, white; ECM glycoproteins, black; ECM-affiliated proteins, blue; proteoglycans, red; ECM regulators, yellow; secreted factors, green).


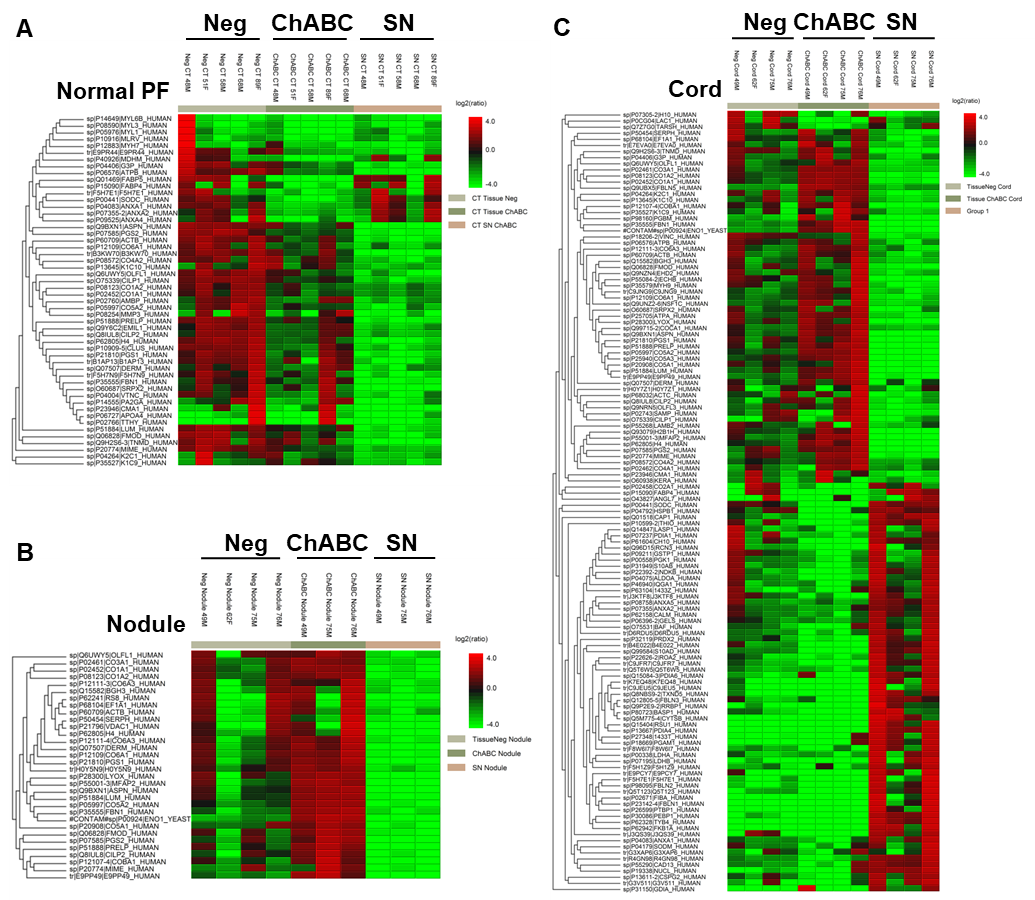


**Figure S3. Evidence for loss of proteins into supernatant with chondroitinase ABC treatment.** Heatmap of relative protein abundance in untreated (Neg) and chondroitinase ABC-treated (ChABC) samples compared to ChABC supernatant (SN) each normalised to equivalent tissue weight (*n* = 4 except for ChABC-treated nodule for which *n* = 3). (A) Normal PF. (B) Dupuytren’s nodule. (C) Dupuytren’s cord.


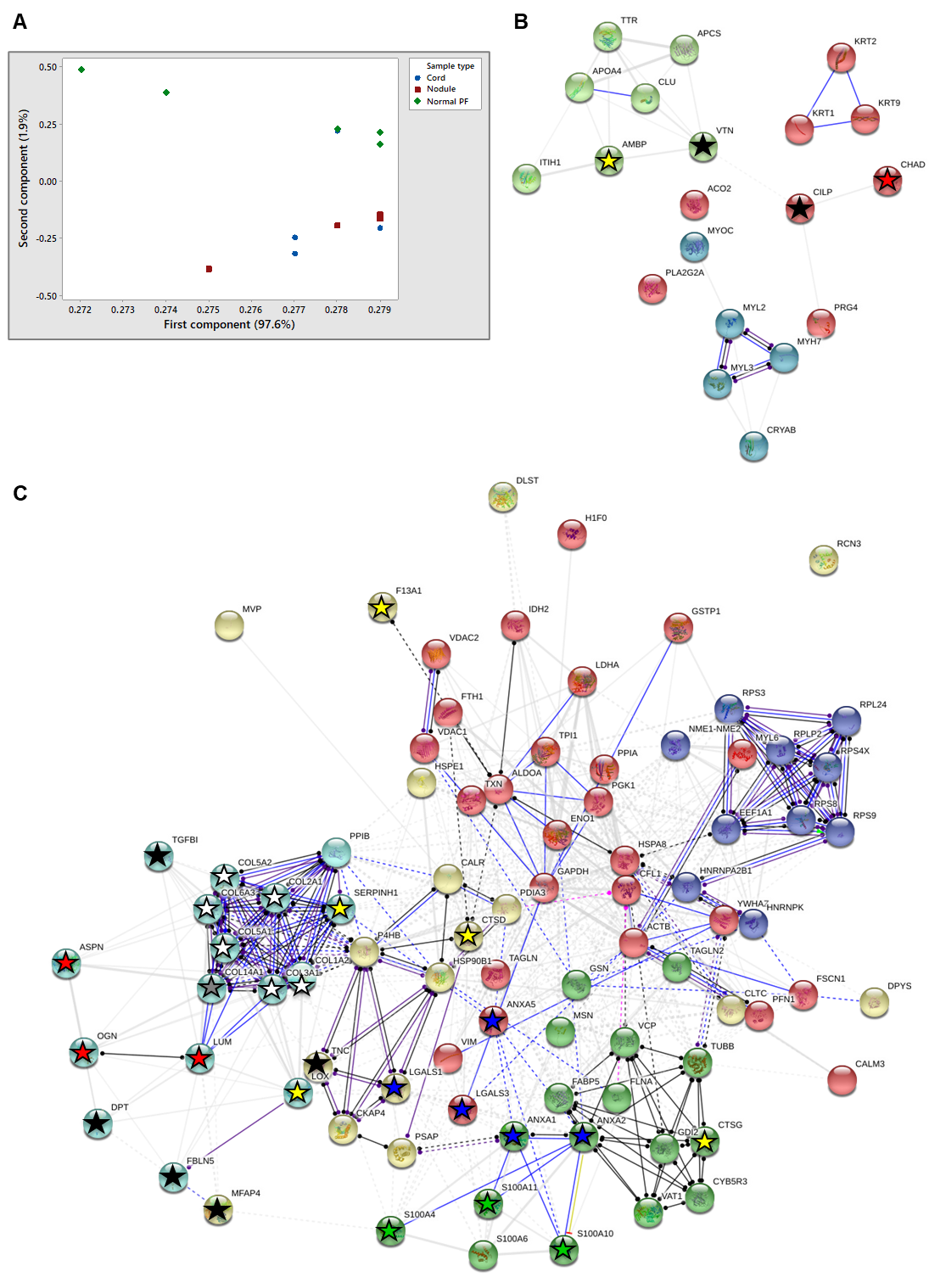


**Figure S4. Label-free proteomics analysis of normal PF and Dupuytren’s explant tissue processed without chondroitinase ABC treatment.** (A) PCA score plot grouped by tissue type. (B) STRING interaction network for proteins enriched in normal PF explant tissue. (C) STRING interaction network for proteins enriched in Dupuytren’s explant tissue. Circle colours indicate k-means clusters of proteins. Line colours indicate activation (green), binding (blue), phenotype (cyan), reaction (black), inhibition (red), catalysis (purple), post-translational modification (pink), or transcriptional regulation (yellow). Line ends indicate positive (arrowhead), negative (orthogonal line), or unspecified effect (circle). Matrisomal proteins are indicated on the STRING diagrams with a star (collagens, white; ECM glycoproteins, black; ECM-affiliated proteins, blue; proteoglycans, red; ECM regulators, yellow; secreted factors, green).


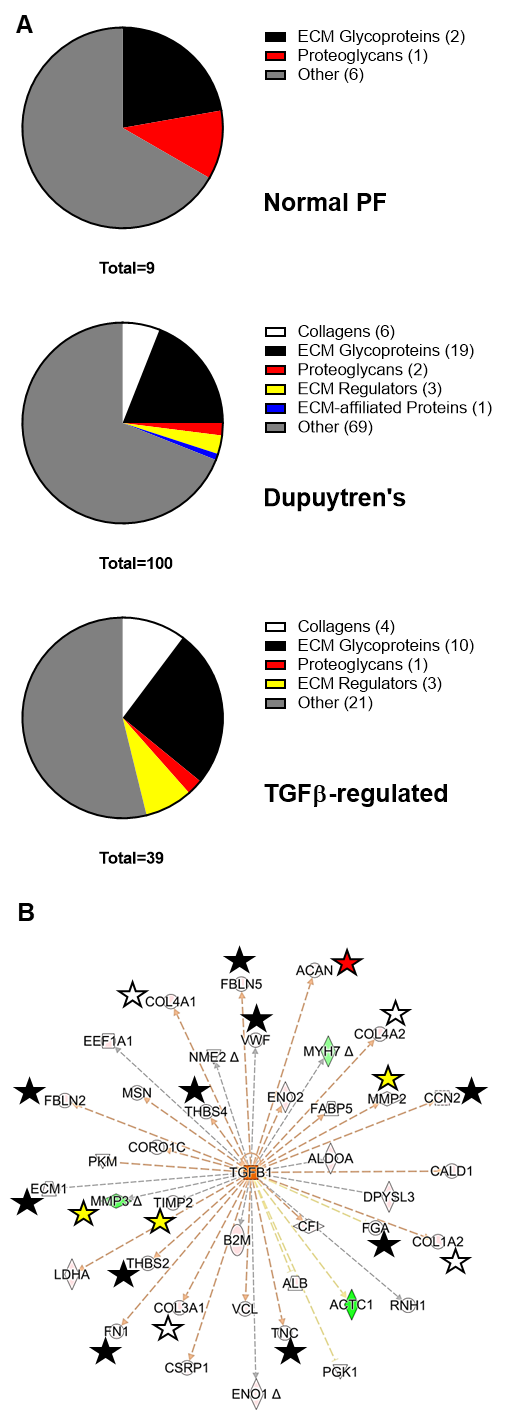


**Figure S5. Matrisomal enrichment in normal PF and Dupuytren’s explant media and upstream regulator analysis.** (A) Enriched proteins in normal PF media, Dupuytren’s media, and those in Dupuytren’s media predicted to be regulated by TGFβ, subdivided based on matrisomal classification. (B) Ingenuity Pathway Analysis diagram highlighting matrisomal proteins within those predicted to be regulated by TGFβ. Red: increased abundance; green: decreased abundance; orange: predicted to lead to activation; blue: predicted to lead to inhibition; yellow: findings inconsistent with state of downstream molecule: gray: effect not predicted. Matrisomal proteins are indicated by a star (collagens, white; extracellular matrix (ECM) glycoproteins, black; ECM-affiliated proteins, blue; proteoglycans, red; ECM regulators, yellow; secreted factors, green).


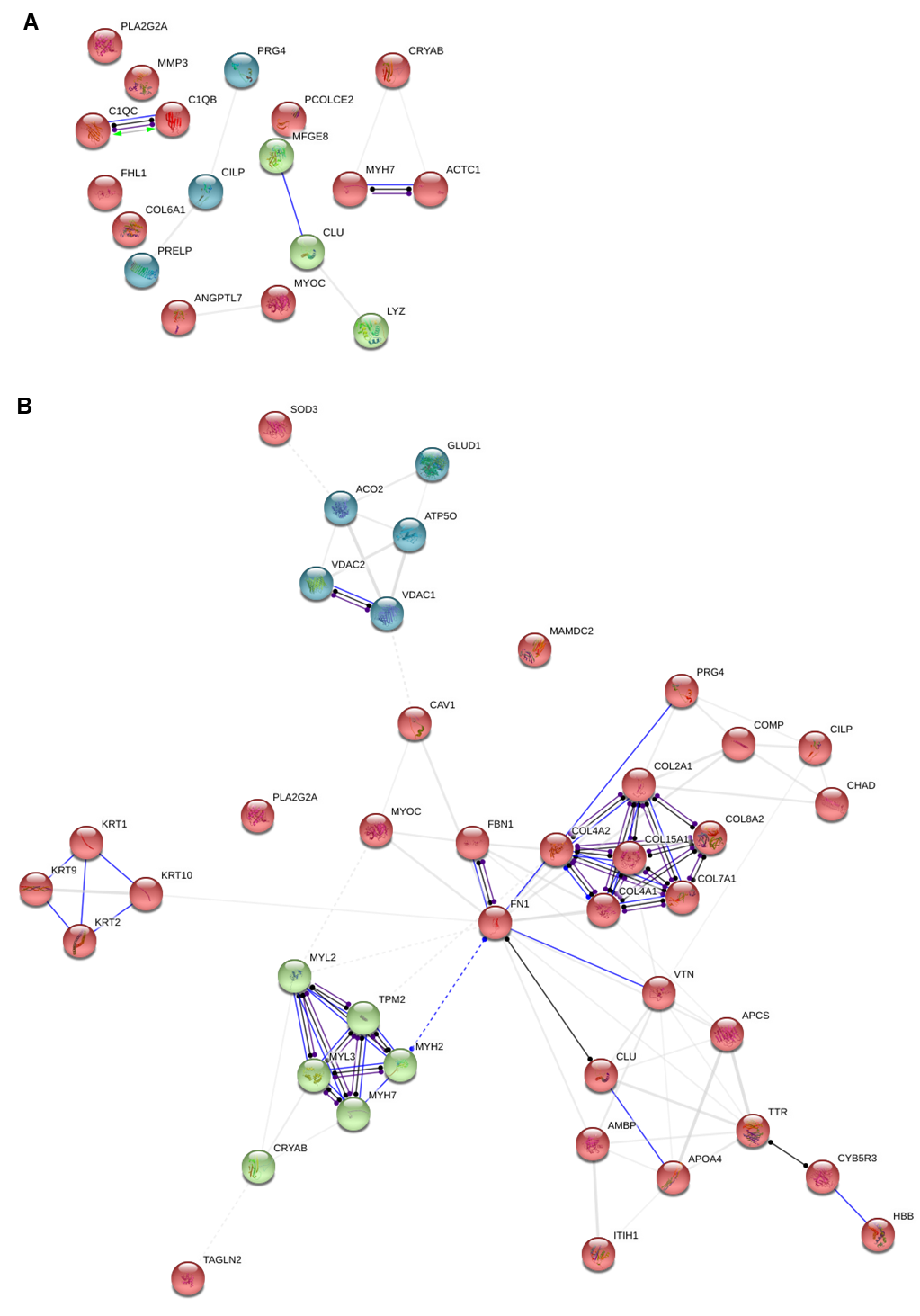


**Figure S6. STRING interaction networks for normal PF and Dupuytren’s explant proteomes from media normalised using total ion chromatogram.** (A) Interaction network for proteins enriched in normal PF explant media. (B) Interaction network for proteins enriched in Dupuytren’s explant media. Circle colours indicate k-means clusters of proteins. Line colours indicate activation (green), binding (blue), phenotype (cyan), reaction (black), inhibition (red), catalysis (purple), post-translational modification (pink), or transcriptional regulation (yellow). Line ends indicate positive (arrowhead), negative (orthogonal line), or unspecified effect (circle).


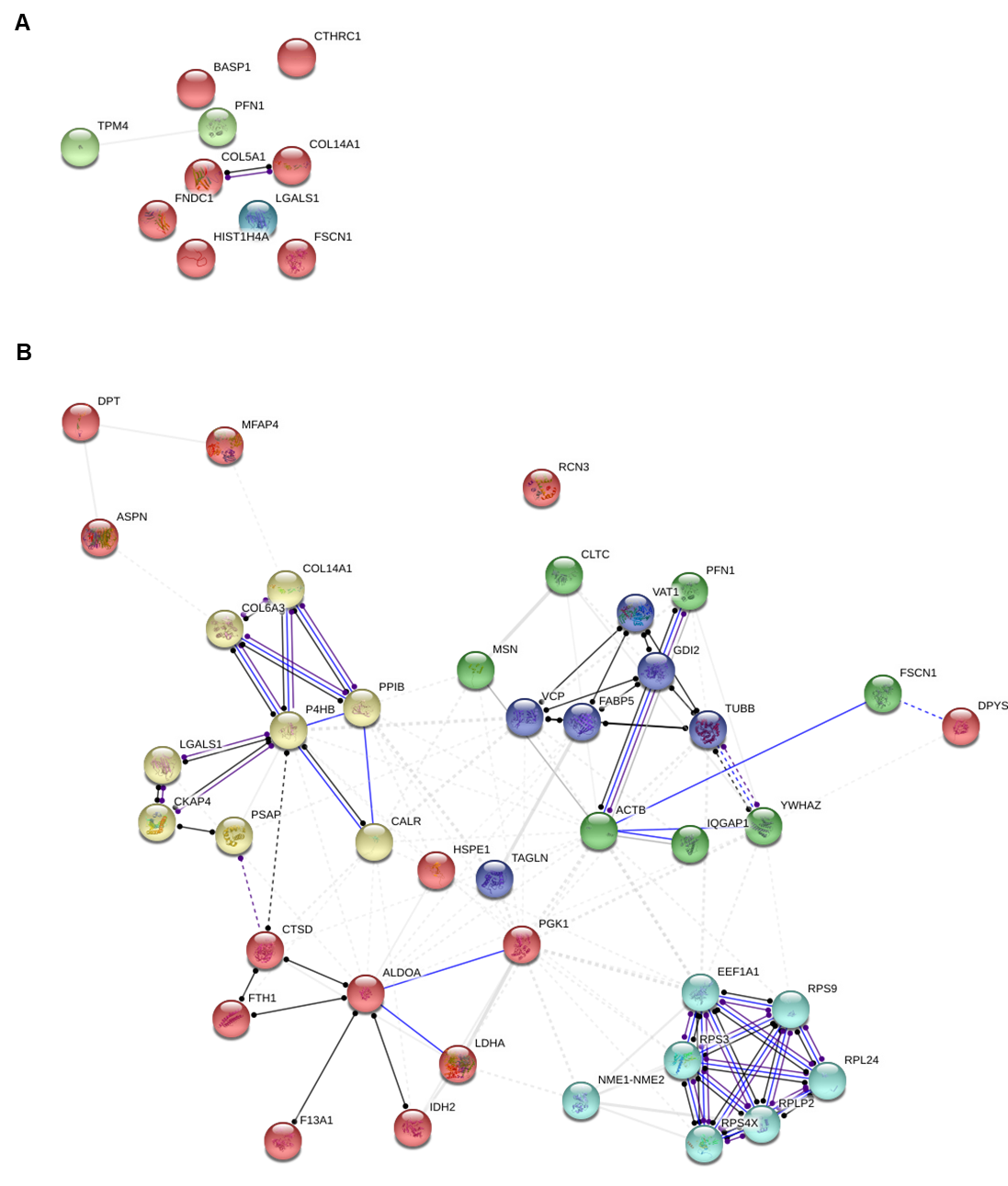


**Figure S7. STRING interaction networks for normal PF and Dupuytren’s explant proteomes from tissue normalised using total ion chromatogram.** (A) Interaction network for proteins enriched in normal PF tissue. (B) Interaction network for proteins enriched in Dupuytren’s tissue. Circle colours indicate k-means clusters of proteins. Line colours indicate activation (green), binding (blue), phenotype (cyan), reaction (black), inhibition (red), catalysis (purple), post-translational modification (pink), or transcriptional regulation (yellow). Line ends indicate positive (arrowhead), negative (orthogonal line), or unspecified effect (circle).


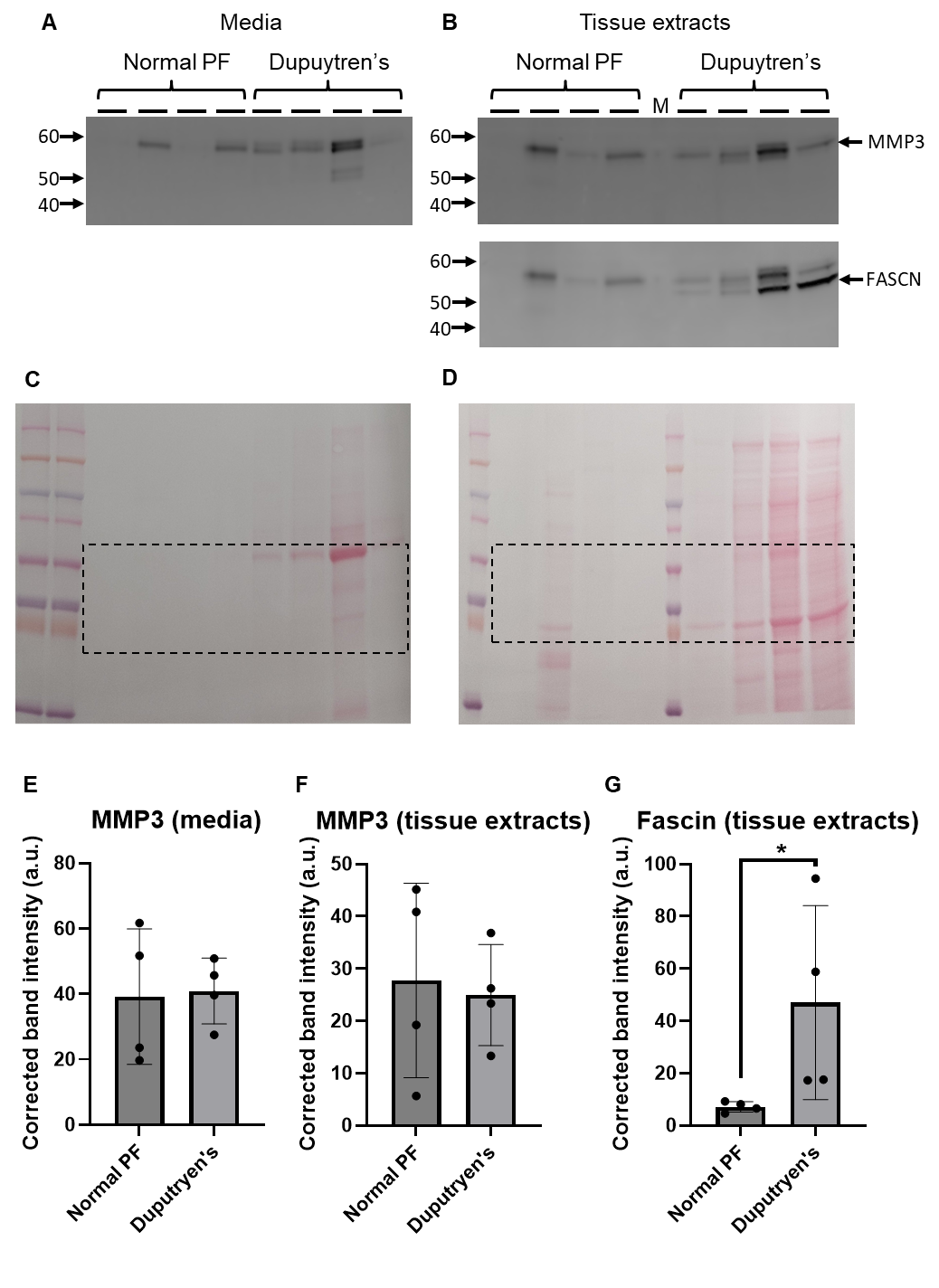


**Figure S8. Western blotting analysis of selected proteins identified using proteomics.** (A) Image of western blot membrane after probing normal PF and Dupuytren’s media samples with antibody against MMP3. (B) Image of western blotting membrane after probing normal PF and Dupuytren’s tissue extracts with antibody against MMP3 and then re-probing with antibody against fascin (FASCN). (C and D) Ponceau S-stained membrane of (C) media samples or (D) tissue extracts. (E–G) Relative quantification of MMP3 in (E) media or (F) tissue extracts or (G) fascin in tissue extracts. *n*= 4 for both normal PF and Dupuytren’s. Sample details are provided in Table 1.


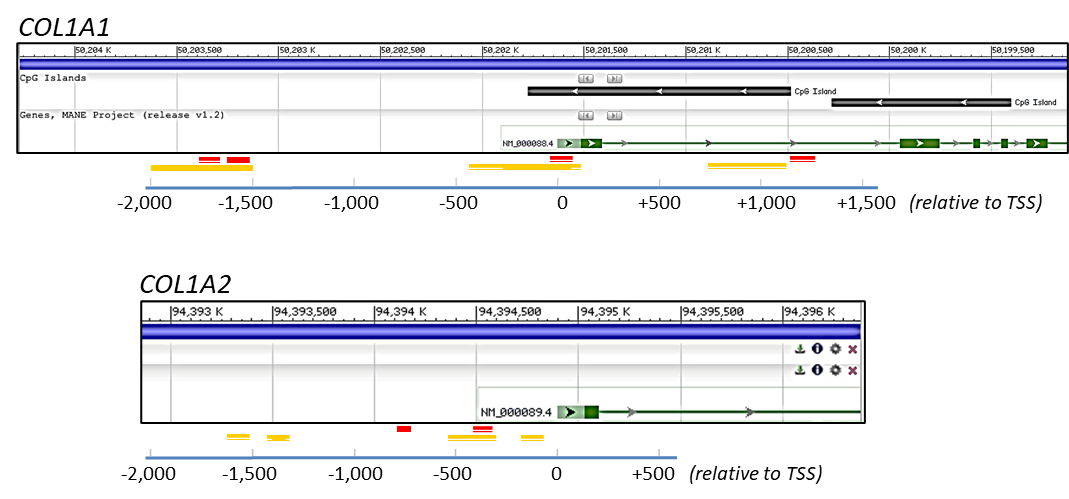


**Figure S9. Locations of regions analysed by pyrosequencing in *COL1* genes**. The map of the *COL1* genes (shaded blue bar, Homo sapiens chromosome 17, GRCh38.p14 Primary Assembly for *COL1A1* and chromosome 7, GRCh38.p14 for *COL1A2*, from <https://www.ncbi.nlm.nih.gov/gene/>, date last accessed 4 December 2025) shows CpG islands (black) and transcripts (green). Regions analysed by pyrosequencing in this study are indicated by compound red lines, with regions showing methylation > 10% being filled. Exact locations of individual CpG sites are shown in the supplementary material, Table S3. Human *COL1* regions analysed in previous studies are shown, with compound yellow for *COL1A1* [84] and *COL1A2* [86] and regions with >10% methylation being filled. The distance from the transcription start site (TSS) for each gene is indicated by a blue line.


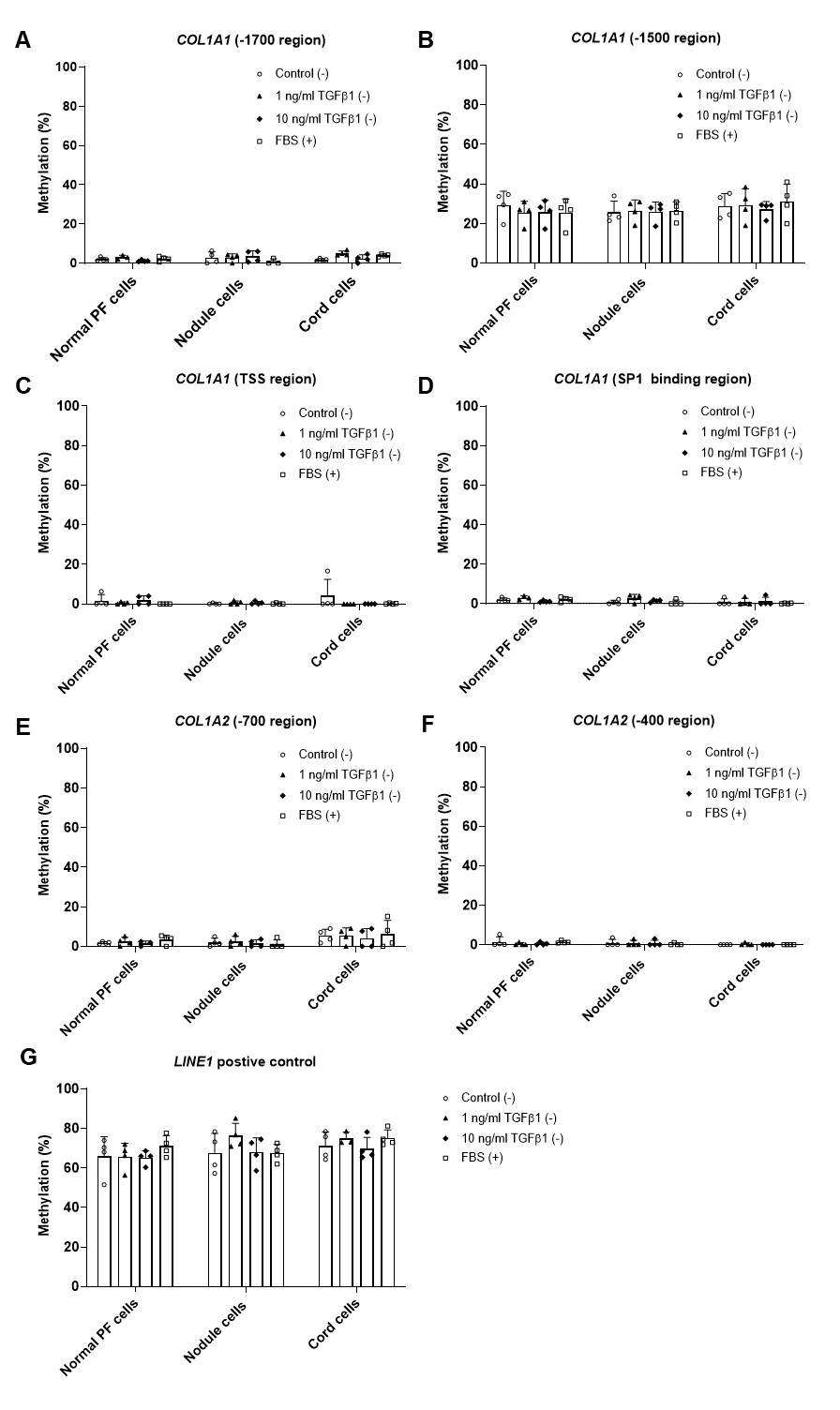


**Figure S10. TGFβ treatment does not affect CpG methylation in human *COL1* regulatory regions**. Average methylation (%) across CpG sites after treatment with control, 1 ng/ml, or 10 ng/ml TGFβ1 treatment in serum-free conditions (−), or with 10% FBS (+) in (A) *COL1A1* (−1700 region), (B) *COL1A1* (−1500 region), (C) *COL1A1* transcriptional start site (TSS) region, (D) *COL1A1* SP1 binding site region, (E) *COL1A2* (−700 region), (F) *COL1A2* (−400 region), and in (G) the *LINE1* positive control region. Individual data points represent biological replicates (*n*= 4). No significant differences were identified. Sample details are given in Table 1.


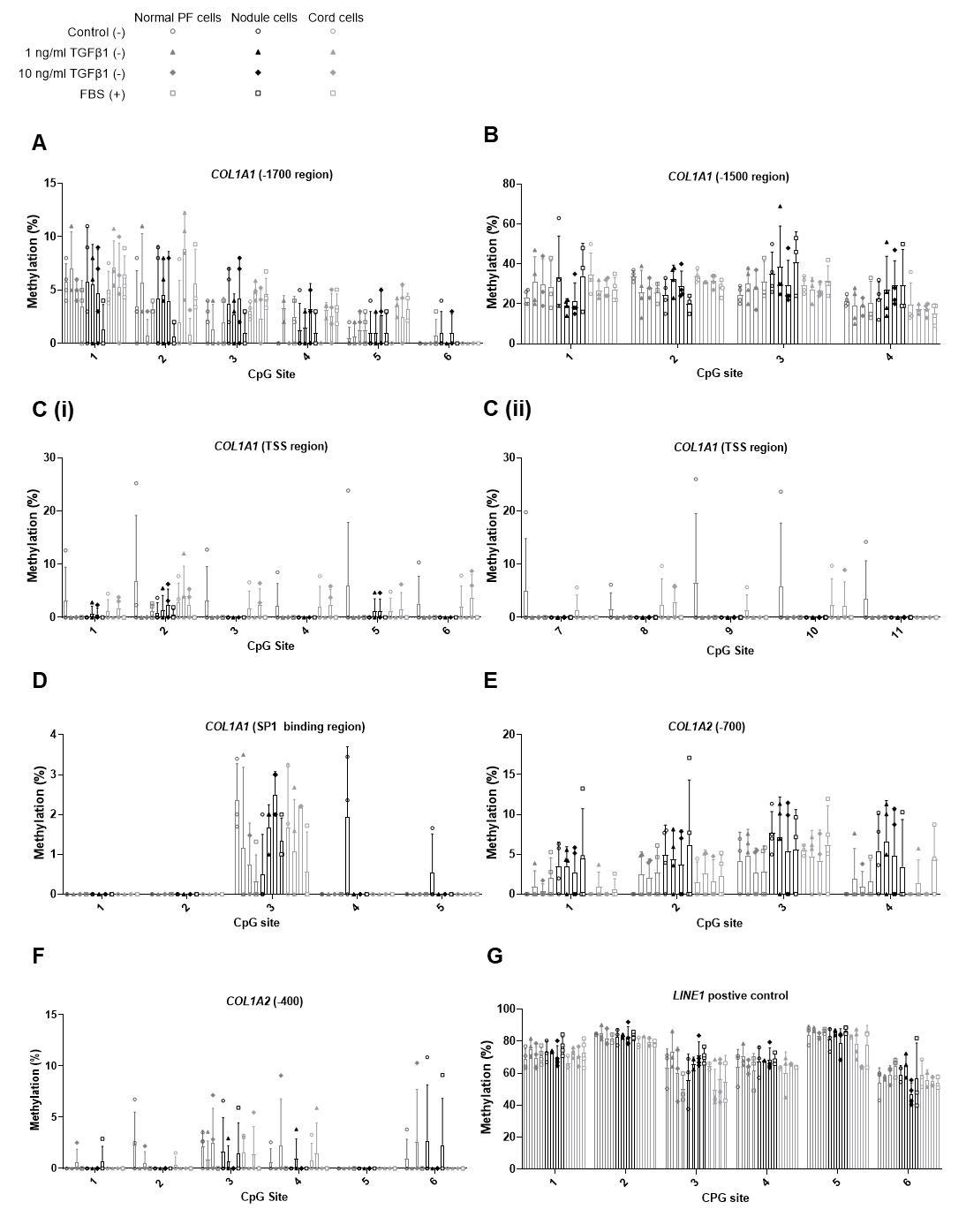


**Figure S11. Methylation levels at individual CpG sites in human *COL1* regulatory regions with TGFβ1 treatment.** Methylation (%) at specific CpG sites after treatment with control, 1 ng/ml, or 10 ng/ml TGFβ1 treatment in serum-free conditions (−), or with 10% FBS (+) in (A) COL1A1 (−1700 region) (six sites), (B) *COL1A1* (−1500 region) (four sites), (C) *COL1A1* transcriptional start site (TSS) region (11 sites), (D) *COL1A1* (SP1 binding site region) (five sites), (E) *COL1A2* (−700 region) (four sites), (F) *COL1A2* (−400 region) (six sites), and (G) *LINE1* positive control region (six  sites). Individual data points represent biological replicates (*n* = 4). No significant differences were identified. Locations of individual CpG sites are given in the supplementary material, Table S3. Sample details are provided in Table 1.

**Figure S12. Comparison of amount of labelled collagen in media of tendon-like constructs treated with TGFβ1 or inhibitor SD208.** Densitometric quantification of the relative amounts of radiolabelled (pro)collagen present in conditioned media from normal PF, Dupuytren’s nodule, and Dupuytren’s cord cells (*n* = 4) after treatment with TGFβ1 or SD208 in serum-free conditions (−) as compared to vehicle control treatments. Data are derived from Figure 5F,I. ****p* < 0.001.

## **Supplementary tables**

**Table S1.** Primer sequences used for RT-qPCR.

| **Species** | **Gene** | **Forward (5′-3′)** | **Reverse (5′-3′)** | **Source** |
| --- | --- | --- | --- | --- |
| Human | *COL1A1* | GTTCAGCTTTGTGGACCTCCG | GATTGGTGGGATGTCTTCGTCT | Designed [18] |
|  | *COL1A2* | GCCTGCCCTTCCTTGATATT | TGAAACAGACTGGGCCAATG | [87] |
|  | *GAPDH* | ATGGGGAAGGTGAAGGTCG | TAAAAGCAGCCCTGGTGACC | Designed [18] |
| Equine | *COL1A1* | CATGTTCAGCTTTGTGGACCT | TGACTGCTGGGATGTCTTCTT | Designed [18] |
|  | *COL1A2* | AGGAGTCTGCATGTCTAAGTGG | TTTCCTGCAGTTGCCTCTTGT | Designed |
|  | *GAPDH* | GCATCGTGGAGGGACTCA | GCCACATCTTCCCAGAGG | Designed [18] |

**Table S2.** Primer sequences for pyrosequencing analysis.

| **Gene/region** | **Forward (* biotinylated)** | **Reverse (* biotinylated)** | **Sequencing** |
| --- | --- | --- | --- |
| *COL1A1* −1700 | GAATAGAAGGGGAGGGAGT (*) | CCCTTTAATTATAACCCCTACA | TAACCCCTACAATCTCC |
| *COL1A1* −1500 | GGAATTTTGGATGGTTTGG (*) | CCTATTCATTTCTCTCCCTCCT | TTCTCTCCCTCCTATACAACT |
| *COL1A1* TSS | GTTATTAGGATAGTATAAAAGGGGT | AAACCCTAAACATATAAACTCTT (*) | TATTAGGATAGTATAAAAGG |
| *COL1A1* SP1 | TGAGGTATATTTTAAGTTTTGGG (*) | AAAATCCAACCCTCATCC | AAATCCAACCCTCATC |
| *COL1A2* −700 | TTGTTGGTAGGTGGGTTG (*) | AAAAACTTCCCCAAATAACTC | AGGTGGGTTGGGGAGTA |
| *COL1A2* −400 | AAGTTTGGAAATATTATGGGA | CCTACAAATAACCTACAAAACAC (*) | TTGGAAATATTATGGGAT |
| *LINE 1* | TAGGGAGTGTTAGATAGTGGG (*) | CTTCCCAAATAAAACAATACC | CCAAATAAAACAATACCTC |

**Table S3.** Location of analysed COL1 CpG sites on chromosomal assemblies and transcripts.

| **Gene/region** | **Chromosomal location^1^** | **Transcript location^2^** |
| --- | --- | --- |
| *COL1A1 −1700* | 50203368 | −1734 |
| *COL1A1 −1700* | 50203363 | −1729 |
| *COL1A1 −1700* | 50203344 | −1710 |
| *COL1A1 −1700* | 50203336 | −1702 |
| *COL1A1 −1700* | 50203327 | −1693 |
| *COL1A1 −1700* | 50203325 | −1691 |
| *COL1A1 −1500* | 50203204 | −1570 |
| *COL1A1 −1500* | 50203194 | −1560 |
| *COL1A1 −1500* | 50203186 | −1552 |
| *COL1A1 −1500* | 50203165 | −1531 |
| *COL1A1 TSS* | 50201647 | −13 |
| *COL1A1 TSS* | 50201638 | −4 |
| *COL1A1 TSS* | 50201635 | −1 |
| *COL1A1 TSS* | 50201626 | 8 |
| *COL1A1 TSS* | 50201612 | 22 |
| *COL1A1 TSS* | 50201606 | 28 |
| *COL1A1 TSS* | 50201592 | 42 |
| *COL1A1 TSS* | 50201590 | 44 |
| *COL1A1 TSS* | 50201575 | 59 |
| *COL1A1 TSS* | 50201567 | 67 |
| *COL1A1 TSS* | 50201563 | 71 |
| *COL1A1 SP1* | 50200439 | 1195 |
| *COL1A1 SP1* | 50200419 | 1215 |
| *COL1A1 SP1* | 50200413 | 1221 |
| *COL1A1 SP1* | 50200388 | 1246 |
| *COL1A1 SP1* | 50200384 | 1250 |
| *COL1A2 −700* | 94394114 | −780 |
| *COL1A2 −700* | 94394136 | −758 |
| *COL1A2 −700* | 94394155 | −739 |
| *COL1A2 −700* | 94394172 | −722 |
| *COL1A2 −400* | 94394485 | −409 |
| *COL1A2 −400* | 94394511 | −383 |
| *COL1A2 −400* | 94394514 | −380 |
| *COL1A2 −400* | 94394540 | −354 |
| *COL1A2 −400* | 94394558 | −336 |
| *COL1A2 −400* | 94394561 | −333 |

^1^ For *COL1A1*: Homo sapiens chromosome 17, GRCh38.p14 Primary Assembly. For *COL1A2*: Homo sapiens chromosome 7, GRCh38.p14 Primary Assembly.

^2^ For *COL1A1*: NM_000088.4. For *COL1A2* NM_000089.4

**Table S4.** Table of *p* values and statistical tests. Peptide sequences are indicated by amino acid code.

| **Figure** | **Comparison** | **Indication on graph** | ***P* value** | **Method (*post hoc* test)** |
| --- | --- | --- | --- | --- |
| 1A | NPF versus Nodule | * | 0.022 | Two-way ANOVA (Holm–Sidak) |
| 1A | NPF versus Cord | * | 0.023 | Two-way ANOVA ((Holm–Sidak) |
| 1B | NPF versus Nodule | ** | 0.008 | Two-way ANOVA (Holm–Sidak) |
| 1B | NPF versus Cord | * | 0.015 | Two-way ANOVA (Holm–Sidak) |
| 1C | Peptides by tissue type | * | 0.047 | One-way ANOVA on ranks (Dunn's uninformative) |
| 1D | VLCDDVICDETK versus DVWKPEPCR | ** | 0.002 | Two-way ANOVA (Holm–Sidak) |
| 1F | Tissue type | * | 0.048 | Two-way ANOVA (Holm–Sidak uninformative) |
| 1F | QYNVGPSVERSUSK versus YEKPGSPPR | * | 0.029 | Two-way ANOVA (Holm–Sidak) |
| 1G | NPF versus Nodule | *** | < 0.001 | Two-way ANOVA (Holm–Sidak) |
| 1G | NPF versus Cord | *** | < 0.001 | Two-way ANOVA (Holm–Sidak) |
| 1H | Peptides by tissue type | * | 0.012 | One-way ANOVA on ranks (Dunn's uninformative) |
| 1I | NPF versus Nodule | *** | 0.001 | Two-way ANOVA (Holm–Sidak) |
| 1I | NPF versus Cord | * | 0.031 | Two-way ANOVA (Holm–Sidak) |
| 1J | NPF: TYFFVEDK versus Nodule IVNYTPD | ** | 0.002 | One-way ANOVA on ranks (Dunn's) |
| 1J | NPF: TYFFVEDK versus Cord IVNYTPDLPK | ** | 0.002 | One-way ANOVA on ranks (Dunn's) |
| 1J | NPF: NSMEPGFPK versus Nodule IVNYTPD | * | 0.011 | One-way ANOVA on ranks (Dunn's) |
| 1J | NPF: NSMEPGFPK versus Cord IVNYTPDLPK | * | 0.011 | One-way ANOVA on ranks (Dunn's) |
| 1L | NPF versus Nodule | ** | 0.006 | Two-way ANOVA (Holm–Sidak) |
| 3A | 0.5 h Aged 10 ng/ml TGFB versus Young serum free | None | 0.006 | Repeated measures two-way ANOVA |
| 3A | 0.5 h Aged 10 ng/ml TGFB versus Young 1 ng/ml TGFB | None | 0.005 | Repeated measures two-way ANOVA |
| 3A | 0.5 h Aged 10 ng/ml TGFB versus Young 10 ng/ml TGFB | None | 0.02 | Repeated measures two-way ANOVA |
| 3A | 0.5 h Aged 10 ng/ml TGFB versus Aged serum free | None | 0.003 | Repeated measures two-way ANOVA |
| 3A | 0.5 h Aged 10 ng/ml TGFB versus Aged 1 ng/ml TGFB | None | 0.001 | Repeated measures two-way ANOVA |
| 3A | 1 h Aged 10 ng/ml TGFB versus Young serum free | None | 0.04 | Repeated measures two-way ANOVA |
| 3A | 1 h Aged 10 ng/ml TGFB versus Young 1 ng/ml TGFB | None | 0.04 | Repeated measures two-way ANOVA |
| 3A | 1 h Aged 10 ng/ml TGFB versus Aged serum free | None | 0.05 | Repeated measures two-way ANOVA |
| 3A | 1 h Aged 10 ng/ml TGFB versus Aged 1 ng/ml TGFB | None | 0.03 | Repeated measures two-way ANOVA |
| 3A | 4 h Aged 10 ng/ml TGFB versus Young serum free | None | 0.01 | Repeated measures two-way ANOVA |
| 3A | 4 h Aged 10 ng/ml TGFB versus Young 1 ng/ml TGFB | None | 0.003 | Repeated measures two-way ANOVA |
| 3A | 4 h Aged 10 ng/ml TGFB versus Aged serum free | None | 0.02 | Repeated measures two-way ANOVA |
| 3A | 4 h Aged 10 ng/ml TGFB versus Aged 1 ng/ml TGFB | None | 0.01 | Repeated measures two-way ANOVA |
| 3A | 4 h old serum free versus Young serum free | None | 0.01 | Repeated measures two-way ANOVA |
| 3A | 24 h Young 10 ng/ml TGFB versus Young serum free | None | 0.006 | Repeated measures two-way ANOVA |
| 3A | 24 h Young 10 ng/ml TGFB versus Young 1 ng/ml TGFB | None | < 0.001 | Repeated measures two-way ANOVA |
| 3A | 24 h Aged serum free versus Young 10 ng/ml TGFB | None | 0.005 | Repeated measures two-way ANOVA |
| 3A | 24 h Aged 1 ng/ml TGFB versus Young 10 ng/ml TGFB | None | 0.04 | Repeated measures two-way ANOVA |
| 3A | 24 h Aged 10 ng/ml TGFB versus Young serum free | None | 0.005 | Repeated measures two-way ANOVA |
| 3A | 24 h Aged 10 ng/ml TGFB versus Young 1 ng/ml TGFB | None | < 0.001 | Repeated measures two-way ANOVA |
| 3A | 24 h Aged 10 ng/ml TGFB versus Aged serum free | None | 0.004 | Repeated measures two-way ANOVA |
| 3A | 24 h Aged 10 ng/ml TGFB versus Aged 1 ng/ml TGFB | None | 0.006 | Repeated measures two-way ANOVA |
| 3A | 48 h Young 10 ng/ml TGFB versus Young serum free | None | 0.009 | Repeated measures two-way ANOVA |
| 3A | 48 h Young 10 ng/ml TGFB versus Young 1 ng/ml TGFB | None | 0.004 | Repeated measures two-way ANOVA |
| 3A | 48 h Aged serum free versus Young serum free | None | 0.008 | Repeated measures two-way ANOVA |
| 3A | 48 h Aged serum free versus Young 10 ng/ml TGFB | None | 0.01 | Repeated measures two-way ANOVA |
| 3A | 48 h Aged 1 ng/ml TGFB versus Young serum free | None | 0.04 | Repeated measures two-way ANOVA |
| 3A | 48 h Aged 1 ng/ml TGFB versus Aged serum free | None | 0.05 | Repeated measures two-way ANOVA |
| 3A | 48 h Aged 10 ng/ml TGFB versus Young serum free | None | 0.006 | Repeated measures two-way ANOVA |
| 3A | 48 h Aged 10 ng/ml TGFB versus Young 1 ng/ml TGFB | None | 0.002 | Repeated measures two-way ANOVA |
| 3A | 48 h Aged 10 ng/ml TGFB versus Aged serum free | None | 0.006 | Repeated measures two-way ANOVA |
| 3B | 0 hs Aged serum free versus Young serum free | None | 0.05 | Repeated measures two-way ANOVA |
| 3B | 0.5 h Young 1 ng/ml TGFB versus Young serum free | None | < 0.001 | Repeated measures two-way ANOVA |
| 3B | 0.5 h Aged serum free versus Young serum free | None | 0.04 | Repeated measures two-way ANOVA |
| 3B | 1 h Young 1 ng/ml TGFB versus Young serum free | None | < 0.001 | Repeated measures two-way ANOVA |
| 3B | 1 h Aged serum free versus Young serum free | None | 0.002 | Repeated measures two-way ANOVA |
| 3B | 4 h old serum free versus Young serum free | None | 0.001 | Repeated measures two-way ANOVA |
| 3B | 24 h Aged serum free versus Young serum free | None | < 0.001 | Repeated measures two-way ANOVA |
| 3B | 48 h Young 10 ng/ml TGFB versus Young 1 ng/ml TGFB | None | 0.03 | Repeated measures two-way ANOVA |
| 3B | 48 h Aged serum free versus Young serum free | None | < 0.001 | Repeated measures two-way ANOVA |
| 3B | 48 h Aged serum free versus Young 10 ng/ml TGFB | None | 0.03 | Repeated measures two-way ANOVA |
| 3B | 48 h Aged 10 ng/ml TGFB versus Young 10 ng/ml TGFB | None | 0.03 | Repeated measures two-way ANOVA |
| 3C | 0.5 hs Young 1 ng/ml TGFB versus Young serum free | None | 0.05 | Repeated measures two-way ANOVA |
| 3C | 1 h Young 10 ng/ml TGFB versus Young serum free | None | 0.01 | Repeated measures two-way ANOVA |
| 3C | 1 h Young 10 ng/ml TGFB versus Young 1 ng/ml TGFB | None | 0.03 | Repeated measures two-way ANOVA |
| 3C | 1 h Aged serum free versus Young 10 ng/ml TGFB | None | 0.04 | Repeated measures two-way ANOVA |
| 3C | 1 h Aged 10 ng/ml TGFB versus Young serum free | None | 0.05 | Repeated measures two-way ANOVA |
| 3C | 4 h old serum free versus Young serum free | None | 0.01 | Repeated measures two-way ANOVA |
| 3C | 4 h Aged 10 ng/ml TGFB versus Young serum free | None | 0.001 | Repeated measures two-way ANOVA |
| 3C | 4 h Aged 10 ng/ml TGFB versus Young 1 ng/ml TGFB | None | < 0.001 | Repeated measures two-way ANOVA |
| 3C | 4 h Aged 10 ng/ml TGFB versus Aged serum free | None | 0.003 | Repeated measures two-way ANOVA |
| 3C | 4 h Aged 10 ng/ml TGFB versus Aged 1 ng/ml TGFB | None | < 0.001 | Repeated measures two-way ANOVA |
| 3C | 24 h Aged 10 ng/ml TGFB versus Aged 1 ng/ml TGFB | none | 0.05 | Repeated measures two-way ANOVA |
| 3D | Young versus old | *** | < 0.001 | Two-way ANOVA (Holm–Sidak) |
| 3D | SF versus 1 ng/ml TGFB1 | * | 0.024 | Two-way ANOVA (Holm–Sidak) |
| 3D | 1 ng/ml TGFB1 versus 10 ng/ml TGFB1 | ** | 0.004 | Two-way ANOVA (Holm–Sidak) |
| 3D | SF versus 10 ng/ml TGFB1 | *** | < 0.001 | Two-way ANOVA (Holm–Sidak) |
| 3D | 1 ng/ml TGFB2 versus 10 ng/ml TGFB2 | *** | < 0.001 | Two-way ANOVA (Holm–Sidak) |
| 3D | SF versus 10 ng/ml TGFB2 | *** | < 0.001 | Two-way ANOVA (Holm–Sidak) |
| 3D | SF versus 1 ng/ml TGFB3 | * | 0.039 | Two-way ANOVA (Holm–Sidak) |
| 3D | 1 ng/ml TGFB3 versus 10 ng/ml TGFB3 | * | 0.017 | Two-way ANOVA (Holm–Sidak) |
| 3D | SF versus 10 ng/ml TGFB3 | *** | < 0.001 | Two-way ANOVA (Holm–Sidak) |
| 3D | 1 ng/ml TGFB1 versus 10 ng/ml TGFB2 | None | < 0.001 | Two-way ANOVA (Holm–Sidak) |
| 3D | 1 ng/ml TGFB1 versus 10 ng/ml TGFB3 | None | 0.028 | Two-way ANOVA (Holm–Sidak) |
| 3D | 1 ng/ml TGFB2 versus 10 ng/ml TGFB1 | None | < 0.001 | Two-way ANOVA (Holm–Sidak) |
| 3D | 1 ng/ml TGFB2 versus 10 ng/ml TGFB3 | None | 0.004 | Two-way ANOVA (Holm–Sidak) |
| 3D | 1 ng/ml TGFB3 versus 10 ng/ml TGFB1 | None | 0.002 | Two-way ANOVA (Holm–Sidak) |
| 3D | 1 ng/ml TGFB3 versus 10 ng/ml TGFB2 | None | < 0.001 | Two-way ANOVA (Holm–Sidak) |
| 3E | SF (young) versus 10 ng/ml TGFB1 (young) | * | 0.029 | One-way ANOVA on ranks |
| 3E | 10 ng/ml TGFB1 (young) versus 10 ng/ml TGFB1 (old) | None | 0.034 | One-way ANOVA on ranks |
| 3E | 10 ng/ml TGFB1 (young) versus 1 ng/ml TGFB3 (old) | None | 0.014 | One-way ANOVA on ranks |
| 3E | 1 ng/ml TGFB2 (young) versus 1 ng/ml TGFB3 (old) | None | 0.04 | One-way ANOVA on ranks |
| 3F | 10 ng/ml TGFB1 (young) versus 10 ng/ml TGFB1 (old) | * | 0.021 | One-way ANOVA on ranks |
| 3F | 10 ng/ml TGFB1 (old) versus 10 ng/ml TGFB2 (young) | None | 0.048 | One-way ANOVA on ranks |
| 3F | 10 ng/ml TGFB1 (old) versus 10 ng/ml TGFB3 (young) | None | 0.043 | One-way ANOVA on ranks |
| 3F | 10 ng/ml TGFB1 (young) versus 10 ng/ml TGFB3 (old) | None | 0.039 | One-way ANOVA on ranks |
| 3F | 10 ng/ml TGFB1 (old) versus 1 ng/ml TGFB3 (young) | None | 0.041 | One-way ANOVA on ranks |
| 4A | Normal PF cells versus Nodule cells | *** | < 0.001 | Two-way ANOVA (Holm–Sidak) |
| 4A | Normal PF cells versus Cord cells | *** | < 0.001 | Two-way ANOVA (Holm–Sidak) |
| 4A | Control (-) versus 1 ng/ml TGFβ1 (-) | ** | 0.003 | Two-way ANOVA (Holm–Sidak) |
| 4A | Control (-) versus 10 ng/ml TGFβ1 (-) | *** | < 0.001 | Two-way ANOVA (Holm–Sidak) |
| 4A | 1 ng/ml TGFβ1 (-) versus 10 ng/ml TGFβ1 (-) | ** | 0.003 | Two-way ANOVA (Holm–Sidak) |
| 4A | 10 ng/ml TGFβ1 (-) versus FBS (+) | *** | < 0.001 | Two-way ANOVA (Holm–Sidak) |
| 4B | Normal PF cells versus Nodule cells | *** | < 0.001 | Two-way ANOVA (Holm–Sidak) |
| 4B | Normal PF cells versus Cord cells | *** | < 0.001 | Two-way ANOVA (Holm–Sidak) |
| 4B | Nodule cells versus Cord cells | * | 0.015 | Two-way ANOVA (Holm–Sidak) |
| 4B | Control (-) versus 1 ng/ml TGFβ1 (-) | ** | 0.002 | Two-way ANOVA (Holm–Sidak) |
| 4B | Control (-) versus 10 ng/ml TGFβ1 (-) | *** | < 0.001 | Two-way ANOVA (Holm–Sidak) |
| 4B | 1 ng/ml TGFβ1 (-) versus FBS (+) | ** | 0.002 | Two-way ANOVA (Holm–Sidak) |
| 4B | 10 ng/ml TGFβ1 (-) versus FBS (+) | *** | < 0.001 | Two-way ANOVA (Holm–Sidak) |
| 4C | Normal PF cells, Control (-) versus Nodule cells, Control (-) | ** | 0.004 | Two-way ANOVA (Holm–Sidak) |
| 4C | Normal PF cells, Control (-) versus Cord cells, Control (-) | * | 0.016 | Two-way ANOVA (Holm–Sidak) |
| 4C | Normal PF cells, FBS (+) versus Nodule cells, FBS (+) | * | 0.02 | Two-way ANOVA (Holm–Sidak) |
| 4C | Normal PF cells, FBS (+) versus Cord cells, FBS (+) | * | 0.014 | Two-way ANOVA (Holm–Sidak) |
| 4D | FBS, Normal PF media versus 1 | *** | <  0.001 | One-sample *t*-test |
| 4D | 1 ng/ml TGFβ1 (−), Nodule media versus 1 | * | 0.033 | One-sample *t*-test |
| 4D | 10 ng/ml TGFβ1 (−), Nodule media versus 1 | * | 0.036 | One-sample *t*-test |
| 4D | FBS, Nodule media versus 1 | * | 0.042 | One-sample *t*-test |
| 5A | Nodule versus Normal PF | ** | 0.001 | Two-way ANOVA (Holm–Sidak) |
| 5A | Cord versus Normal PF | ** | 0.002 | Two-way ANOVA (Holm–Sidak) |
| 5F | Normal PF | ***** | 0.037 | One-sample *t*-test |
| 6A | SD208 ratio, nodule ratio | (*) | 0.002 | One-sample *t*-test (one-tailed) |
| S8 | Normal PF versus Dupuytren’s | ***** | 0.025 | Welch's *t*-test |
| S12 | TGFβ1 (10 ng/ml) versus SD208 | *** | < 0.001 | Two-way ANOVA (Holm–Sidak) |

**Table S5**. Reactome pathways from STRING.

| **Sample type** | **Normalisation method** | **Pathway** | **FDR** |
| --- | --- | --- | --- |
| Normal PF | Wet weight of tissue | Striated muscle contraction | 0.0040 |
| Dupuytren’s | Wet weight of tissue | Extracellular matrix organisation | 5.67e−06 |
| Dupuytren’s | Wet weight of tissue | Developmental biology | 9.49e−06 |
| Dupuytren’s | Wet weight of tissue | Haemostasis | 1.57e−05 |
| Dupuytren’s | Wet weight of tissue | Innate immune system | 0.0076 |
| Dupuytren’s | Wet weight of tissue | ER-phagosome pathway | 0.0263 |
| Dupuytren’s | Wet weight of tissue | Apoptosis | 0.0263 |
| Dupuytren’s | Wet weight of tissue | Cytokine signalling in immune system | 0.0486 |
| Normal PF | Total ion chromatogram | Collagen degradation | 0.00078 |
| Normal PF | Total ion chromatogram | Developmental biology | 0.0071 |
| Normal PF | Total ion chromatogram | Striated muscle contraction | 0.0088 |
| Normal PF | Total ion chromatogram | Cellular response to stress | 0.0266 |
| Normal PF | Total ion chromatogram | Mitochondrial protein import | 0.0266 |
| Normal PF | Total ion chromatogram | Extracellular matrix organisation | 0.0432 |
| Dupuytren’s | Total ion chromatogram | Developmental biology | 0.00031 |
| Dupuytren’s | Total ion chromatogram | Extracellular matrix organisation | 0.0082 |
| Dupuytren’s | Total ion chromatogram | Haemostasis | 0.0413 |

**Table S6.** Top 10 upstream regulators identified using IPA for media.

| **Upstream regulator** | **Activation z-score** | ***P* value of overlap** |
| --- | --- | --- |
| TGFB1 | 3.732 | 7.69E−17 |
| PRL | 3.136 | 8.49E−07 |
| F2 | 3.086 | 5.17E−07 |
| IL4 | 2.787 | 2.36E−03 |
| HIF1A | 2.77 | 7.20E−07 |
| CCR2 | 2.728 | 5.19E−12 |
| IL15 | 2.635 | 1.46E−07 |
| IL5 | 2.608 | 4.09E−04 |
| TGF BETA (family) | 2.592 | 1.06E−06 |
| IL13 | 2.508 | 5.50E−04 |

IPA, Ingenuity Pathway Analysis.

**Table S7.** Top 10 upstream regulators identified using IPA for tissue.

| **Upstream regulator** | **Activation z-score** | ***P* value of overlap** |
| --- | --- | --- |
| TGFB1 | 3.878 | 2.48E−19 |
| IL5 | 3.162 | 6.85E−07 |
| CCR2 | 3.148 | 8.76E−11 |
| INSULIN (family) | 3.111 | 3.49E−10 |
| SMAD3 | 3.065 | 2.33E−07 |
| IGF2BP1 | 3 | 2.22E−15 |
| NFE2L2 | 2.896 | 5.79E−07 |
| CD38 | 2.804 | 6.56E−07 |
| HIF1A | 2.755 | 5.89E−08 |
| MKNK1 | 2.646 | 3.59E−07 |

IPA, Ingenuity Pathway Analysis.

**Table S8.** Top 10 diseases and biofunctions identified using IPA for media.

| **Category** | ***P* value** |
| --- | --- |
| Cellular movement | 4.44E−24–2.64E−04 |
| Tissue development | 4.23E−17–2.12E−04 |
| Cellular compromise | 5.25E−16–8.61E−10 |
| Inflammatory response | 5.25E−16–2.8E−04 |
| Cancer | 5.09E−15–3.18E−04 |
| Organismal injury and abnormalities | 5.09E−15–3.18E−04 |
| Reproductive system disease | 5.09E−15–3.02E−04 |
| Neurological disease | 7.52E−15–3.18E−04 |
| Connective tissue disorders | 5.28E−13–3.18E−04 |
| Skeletal and muscular disorders | 5.28E−13–3.18E−04 |

IPA, Ingenuity Pathway Analysis.

**Table S9.** Top 10 diseases and biofunctions identified using IPA for tissue.

| **Category** | ***P* value** |
| --- | --- |
| Cellular movement | 1.91E−22–1.75E−04 |
| Cellular assembly and organisation | 6.19E−22–7.03E−05 |
| Cellular function and maintenance | 6.19E−22–1.89E−04 |
| Neurological disease | 2.01E−20–1.89E−04 |
| Organismal injury and abnormalities | 2.01E−20–1.92E−04 |
| Cellular compromise | 8.38E−19–4.29E−05 |
| Inflammatory response | 8.38E−19–1.24E−04 |
| Hereditary disorder | 1.99E−17–1.89E−04 |
| Skeletal and muscular disorders | 1.99E−17–1.88E−04 |
| Dermatological diseases and conditions | 3.53E−17–1.75E−04 |

IPA, Ingenuity Pathway Analysis.

**Table S10.** Number of unique neopeptide sequences in each sample type filtered for occurrence in at least three samples of normal PF, Dupuytren’s nodule, or cord media samples.

|  |  | **Dupuytren's** | |
| --- | --- | --- | --- |
| **Protein name and peptide sequence(s)** | **Normal PF** | **Nodule** | **Cord** |
|  |  |  |  |
| Actin, cytoplasmic 1 (ACTB) | 2 | 8 | 10 |
| DESGPSIVHR |  | 4 | 4 |
|  |  |  |  |
| Adipocyte enhancer-binding protein 1 (AEBP1) |  | 6 | 7 |
| AQNEVVATDDLDFR |  | 3 | 3 |
|  |  |  |  |
| Cartilage intermediate layer protein 1 (CILP) | 20 | 1 | 4 |
| LQSTPAQSPAAGTVQGR | 4 |  |  |
| STPAQSPAAGTVQGR | 3 |  |  |
| VLASLAGEELQAVESSPK | 3 |  |  |
|  |  |  |  |
| Collagen alpha-1(IV) chain (COL4A1) |  | 3 | 1 |
| SLLYVQGNER |  | 3 |  |
|  |  |  |  |
| Collagen alpha-3(VI) chain (COL6A3) | 6 | 3 | 4 |
| ALTETDICK |  | 3 | 1 |
| IGEQGISGPR | 3 |  |  |
|  |  |  |  |
| Complement C3 (C3) | 10 | 7 | 9 |
| VTNPDGSPAYR |  | 3 | 1 |
|  |  |  |  |
| Fibronectin (FN1) | 38 | 40 | 36 |
| ALVCTCYGGSR |  | 3 | 2 |
|  |  |  |  |
| Hemoglobin subunit beta (HBB) | 2 | 3 | 8 |
| GDLSTPDAVMGNPK |  | 3 | 4 |
|  |  |  |  |
| Immunoglobulin heavy constant gamma 1 (IGHG1) | 8 | 14 | 20 |
| GTAALGCLVK |  | 3 | 3 |
| NQVERSUSLTCLVKG |  | 1 | 3 |
|  |  |  |  |
| Immunoglobulin kappa constant (IGKC) | 12 | 14 | 18 |
| VYACEVTH |  | 3 | 3 |
|  |  |  |  |
| Myocilin (MYOC) | 30 | 2 | 2 |
| SVASPNESSCPEQSQAMSVIHNLQR | 3 |  |  |
| TFSVASPNESSCPEQSQAMSVIHNLQR | 3 |  |  |
| TSADATVNFAYDTGTGISK | 4 |  |  |
|  |  |  |  |
| Peptidyl-prolyl cis-trans isomerase FKBP1A (FKBP1A) |  | 3 | 2 |
| GVQVETISPGDGR |  | 3 | 2 |
|  |  |  |  |
| Proteoglycan 4 (PRG4) | 5 |  |  |
| LSDETNICNGKPVDGLTTLR | 4 |  |  |
|  |  |  |  |
| Tenascin-X (TNXB) | 47 | 14 | 22 |
| SITFTTGLEAPR | 3 |  |  |
| TVAQGPFDSFLVQYR | 4 |  |  |
|  |  |  |  |
| Vimentin (VIM) | 23 | 11 | 10 |
| TVETRDGQVINETSQHHDDLE | 3 |  |  |
|  |  |  |  |
| **Grand total** | **593** | **392** | **525** |

For each protein the total number of identified neopeptides across all samples is indicated. The grand total indicates the total number of neopeptide sequences identified for each sample type.

**Table S11.** Number of unique neopeptide sequences in each sample type filtered for occurrence in at least three samples of normal PF, Dupuytren’s nodule, or cord tissue samples**.**

|  |  | **Dupuytren's** | |
| --- | --- | --- | --- |
| **Protein name and peptide sequence(s)** | **Normal PF** | **Nodule** | **Cord** |
|  |  |  |  |
| Cartilage intermediate layer protein 1 (CILP) | 9 |  |  |
| SAEESNGPIYAFENLR | 4 |  |  |
| VLASLAGEELQAVESSPK | 4 |  |  |
|  |  |  |  |
| Collagen alpha-1(VI) chain (COL6A1) | 14 | 6 | 3 |
| YAELLEDAFLK | 4 |  |  |
|  |  |  |  |
| Collagen alpha-2(I) chain (COL1A2) | 37 | 13 | 9 |
| AGAAGPAGPAGPR | 3 |  |  |
| PGPMGLMGPR | 5 |  |  |
|  |  |  |  |
| Collagen alpha-3(VI) chain (COL6A3) | 5 | 10 | 1 |
| SLETAMSFVAR | 3 |  |  |
|  |  |  |  |
| Decorin (DCN) | 13 | 6 | 2 |
| IVIELGTNPLK | 5 |  |  |
| NLHALILVN | 3 |  |  |
|  |  |  |  |
| Fibromodulin (FMOD) | 10 | 1 |  |
| CTVVDVVNFSK | 4 |  |  |
|  |  |  |  |
| Fibulin-1 (FBLN1) | 3 |  |  |
| DVLLEACCADGHR | 3 |  |  |
|  |  |  |  |
| Keratin, type I cytoskeletal 10 (KRT10) | 8 | 1 | 2 |
| ISALEEQLQQIR | 3 |  |  |
|  |  |  |  |
| Vitronectin (VTN) | 4 |  |  |
| SIAQYWLGCPAPGH | 3 |  |  |
|  |  |  |  |
| **Grand total** | **227** | **143** | **59** |

For each protein the total number of identified neopeptides across all samples is indicated. The grand total indicates the total number of neopeptide sequences identified for each sample type.
